# Supplementary material for: “One feels anger to know there is no one to help us!”. Perceptions of mothers of children with Zika virus-associated microcephaly in Caribbean Colombia: A qualitative study
Source: PLoS Negl Trop Dis. 2022 Apr 18;16(4):e0010328. doi: 10.1371/journal.pntd.0010328 (PMC9015150; doi:10.1371/journal.pntd.0010328)
Supplement: S1 Table — (DOCX) [file pntd.0010328.s001.docx]

**Supporting information**

**S1 Table.** Summary of themes, and sub-themes emerging from the interviews

| **Theme** | **Subtheme** |
| --- | --- |
| **Background experiences from women** | Experienced Chikungunya infection in the past, and compared it with ZIKV as the latter provoking *“milder symptoms”* |
|  | Experienced Dengue or Chikungunya infections in the past |
|  | Had to emigrate from her home town due to the armed conflict in Colombia |
| **Knowledge about ZIKV, its transmission and prevention** | Understanding what Zika is and how to prevent it |
|  | Confusing Zika with a bacterium |
|  | Lack of knowledge about ZIKV sexual transmission |
|  | Understanding who’s vulnerable for suffering consequences of ZIKV |
|  | Explaining that ZIKV is not the only cause of microcephaly, as it could be caused by *“contact with cats”* [toxoplasmosis, disease name not mentioned] |
|  | The Internet as the main source of information |
| **Experiences about their ZIKV diagnosis** | ZIKV screening was performed, but results not received |
|  | Lack of access to laboratory diagnosis |
|  | Feel rage because of not knowing if microcephaly was caused by Zika |
| **Experiences about the diagnosis of microcephaly in their babies** | Knew about the child having microcephaly during pregnancy |
|  | Knew about the child having microcephaly after delivery |
|  | Being told that the baby was going to be born death |
|  | At birth, was told that the baby was not going to be able to talk or walk ever |
| **Discussion about abortion** | Termination of pregnancy was offered in some cases |
|  | Termination of pregnancy if microcephaly on the baby was known in early pregnancy |
|  | Pregnancy too advanced when microcephaly in the foetus was found |
|  | Religious beliefs |
|  | Influence of the family on decision making (example: She was offered a termination of pregnancy after diagnosis of microcephaly, but her mother-in-law made her continue with the pregnancy) |
|  | Personal decision/moral values |
|  | Not being an option due to lack of social acceptability |
|  | Termination of pregnancy only acceptable in the case of rape |
| **Women’s empowerment** | Learned several things (i.e. to be stronger) thanks to her microcephalic child |
|  | Understanding the importance of stimulation, carry the child to a day-care centre/school |
|  | Need to personally “improve” and apply for a grant to study at University to maintain future needs |
|  | Learned how to do therapies at home and learned his medications by heart |
| **Hopes** | Her baby could walk, and talk |
|  | Her baby is not so dependent, so she could have another baby |
|  | Her future baby will not have microcephaly |
|  | Graduate in Nursing to help her baby and other babies in the future |
|  | Create an NGO to help other women carrying microcephalic children, to speak up, and ask for help |
|  | Work from home to continue taking care of her baby and earn some money |
