# Supplementary material for: “One feels anger to know there is no one to help us!”. Perceptions of mothers of children with Zika virus-associated microcephaly in Caribbean Colombia: A qualitative study
Source: PLoS Negl Trop Dis. 2022 Apr 18;16(4):e0010328. doi: 10.1371/journal.pntd.0010328 (PMC9015150; doi:10.1371/journal.pntd.0010328)
Supplement: Attachment — Submitted filename: FC PNTD-D-21-00617_reviewer.pdf [file pntd.0010328.s002.pdf]

PLOS Neglected Tropical Diseases

“One feels anger to know there is no one to help us!”: Perceptions of mothers of children with Zika-associated microcephaly in Caribbean Colombia: A qualitative study

--Manuscript Draft--

**Manuscript Number:**	PNTD-D-21-00617
**Full Title:**	“One feels anger to know there is no one to help us!”: Perceptions of mothers of children with Zika-associated microcephaly in Caribbean Colombia: A qualitative study
**Short Title:**	Perceptions of mothers of children with Zika-associated microcephaly in Colombia
**Article Type:**	Research Article
**Keywords:**	Views; perceptions, attitudes; Zika; microcephaly; Pregnancy; qualitative, grounded theory, Colombia
**Abstract:**	Background<br>The epidemic of Zika virus (ZIKV) in the Americas was associated with a sudden and unprecedented increase in infants born with microcephaly. Colombia was the second most affected country by the epidemic in the region. Primary caregivers of children with ZIKV-associated microcephaly, their mothers mainly, were at higher risk to suffer from anxiety and depression . Often, these women were stigmatized and abandoned by their partners, relatives and communities.<br>Methodology/Principal findings<br>This study aimed to understand the perceptions about ZIKV infection among mothers of children born with microcephaly during the ZIKV epidemic in the Caribbean Colombia, and the barriers and facilitators affecting child health follow up. An exploratory qualitative study, based on Phenomenology and Grounded Theory, was conducted in the Caribbean Colombia. Data were collected through In-Depth Interviews (IDI) from women who delivered a baby with microcephaly during the ZIKV epidemic at Clínica Salud Social, Sincelejo, Sucre District. Themes that emerged during the interviews included experiences from their lives before pregnancy; knowledge about ZIKV; experiences and perceptions when diagnosed; considering a possible termination of pregnancy, and children’s clinical follow up. In some cases, women reported having been told they were having a baby with microcephaly, but decided not to terminate the pregnancy; while in other cases, women found out about their newborn’s microcephaly condition only at birth. The main barriers encountered by participants during children follow up included the lack of psychosocial and economic support, the stigmatization and abandonment by some partners and relatives, and the frustration of seeing the impaired development of their children.<br>Conclusions<br>This study contributed to identifying social, medical, psychological and economic gaps of families with children heavily affected by the ZIKV epidemics. Addressing these gaps is essential to ensure access to quality health care by affected children and their families.
**Additional Information:**	
**Question**	**Response**
**Financial Disclosure**<br><br>Enter a financial disclosure statement that describes the sources of funding for the work included in this submission. Review the submission guidelines for detailed requirements. View published research articles from *PLOS NTDs* for specific examples.<br><br><br>This statement is required for submission	This study was funded by the Government of Spain under grant agreement number PI16/0123, ISCIII-AES Proyectos de Investigación en Salud, 2016; granted to A.B. Additionally, E. M-C. is supported by a predoctoral fellowship from “la Caixa” Foundation (ID 100010434). The fellowship code is LCF/BQ/ES17/11600006. E.M-C. received a mobility grant by Universitat de Barcelona for her stay in Colombia. A.B. is supported by the Ministry of Science, Innovation, and Universities, Government of Spain through a Ramon y Cajal Grant (RYC-2013-14512). ISGlobal is a member of the CERCA Programme, Generalitat de Catalunya.<br><br>The funders had no role in study design, data collection and analysis, decision to publish, or preparation of the manuscript.

Powered by Editorial Manager® and ProduXion Manager® from Aries Systems Corporation

and **will appear in the published article** if the submission is accepted. Please make sure it is accurate.<br><br>**Unfunded studies**<br><br>Enter: *The author(s) received no specific funding for this work.*<br><br>**Funded studies**<br><br>Enter a statement with the following details:<br>• Initials of the authors who received each award<br>• Grant numbers awarded to each author<br>• The full name of each funder<br>• URL of each funder website<br>• Did the sponsors or funders play any role in the study design, data collection and analysis, decision to publish, or preparation of the manuscript?<br>• **NO** - Include this sentence at the end of your statement: *The funders had no role in study design, data collection and analysis, decision to publish, or preparation of the manuscript.*<br>• **YES** - Specify the role(s) played.<br><br>* typeset	
**Competing Interests**<br><br>Use the instructions below to enter a competing interest statement for this submission. On behalf of all authors, disclose any competing interests that could be perceived to bias this work—acknowledging all financial support and any other relevant financial or non-financial competing interests.<br><br>This statement **will appear in the published article** if the submission is accepted. Please make sure it is	The authors have declared that no competing interests exist.

accurate. View published research articles from *PLOS NTDs* for specific examples.<br><br>**NO authors have competing interests**<br><br>Enter: *The authors have declared that no competing interests exist.*<br><br>**Authors with competing interests**<br><br>Enter competing interest details beginning with this statement:<br><br>*I have read the journal's policy and the authors of this manuscript have the following competing interests: [insert competing interests here]*<br><br>* typeset<br><br>This statement is required for submission and **will appear in the published article** if the submission is accepted. Please make sure it is accurate and that any funding sources listed in your Funding Information later in the submission form are also declared in your Financial Disclosure statement.	
**Data Availability**<br><br>Authors are required to make all data underlying the findings described fully available, without restriction, and from the time of publication. PLOS allows rare	No - some restrictions will apply

exceptions to address legal and ethical concerns. See the PLOS Data Policy and FAQ for detailed information.<br><br>A Data Availability Statement describing where the data can be found is required at submission. Your answers to this question constitute the Data Availability Statement and **will be published in the article**, if accepted.<br><br>**Important:** Stating 'data available on request from the author' is not sufficient. If your data are only available upon request, select 'No' for the first question and explain your exceptional situation in the text box.<br><br>Do the authors confirm that all data underlying the findings described in their manuscript are fully available without restriction?	
**Describe where the data may be found in full sentences. If you are copying our sample text, replace any instances of XXX with the appropriate details.**<br><br>• If the data are **held or will be held in a public repository**, include URLs, accession numbers or DOIs. If this information will only be available after acceptance, indicate this by ticking the box below. For example: *All XXX files are available from the XXX database (accession number(s) XXX, XXX.*).<br>• If the data are all contained **within the manuscript and/or Supporting Information files**, enter the following: *All relevant data are within the manuscript and its Supporting Information files.*<br>• If neither of these applies but you are	All relevant data are within the manuscript and its Supporting Information files.

able to provide **details of access elsewhere**, with or without limitations, please do so. For example:<br><br>*Data cannot be shared publicly because of [XXX]. Data are available from the XXX Institutional Data Access / Ethics Committee (contact via XXX) for researchers who meet the criteria for access to confidential data.*<br><br>*The data underlying the results presented in the study are available from (include the name of the third party and contact information or URL).*<br>• This text is appropriate if the data are owned by a third party and authors do not have permission to share the data.<br><br>* typeset	
Additional data availability information:	Tick here if the URLs/accession numbers/DOIs will be available only after acceptance of the manuscript for publication so that we can ensure their inclusion before publication.

Powered by Editorial Manager® and ProduXion Manager® from Aries Systems Corporation

Manuscript

Click here to access/download;Manuscript;3_QUAZIK_Col_PlosNTD_vF.do

"One feels anger to know there is no one to help us!": Perceptions of mothers of children with Zika-associated microcephaly in Caribbean Colombia: A qualitative study

Elena Marbán-Castro[1*], Cristina Enguita-Fernàndez[1,2], Kelly Carolina Romero-Acosta[3], Germán J. Arrieta[3,4,5], Anna Marín-Cos[1], Salim Mattar[4,5], Clara Menéndez[1,2,6], Maria Maixenchs[1,2] and Azucena Bardají[1,2,6]

[1] ISGlobal, Hospital Clínic - Universitat de Barcelona, Barcelona, Spain

[2.] Consorcio de Investigación Biomédica en Red de Epidemiología y Salud Pública (CIBERESP), Madrid, Spain

[3] Corporación Universitaria del Caribe (CECAR), Group of Public Health, Sincelejo, Sucre, Colombia

[4] Clínica Salud Social, Sincelejo, Sucre, Colombia

[5] Universidad de Córdoba - Instituto de investigaciones biológicas del Trópico, Universidad de Córdoba, Montería, Córdoba, Colombia

[6] Centro de Investigação em Saúde de Manhiça (CISM), Maputo, Mozambique

*Corresponding author: elena.marban@isglobal.org

Abstract

Background

The epidemic of Zika virus (ZIKV) in the Americas was associated with a sudden and unprecedented increase in infants born with microcephaly. Colombia was the second most affected country by the epidemic in the region. Primary caregivers of children with ZIKV-associated microcephaly, their mothers mainly, were at higher risk to suffer from anxiety and depression . Often, these women were stigmatized and abandoned by their partners, relatives and communities.

Methodology/Principal findings

This study aimed to understand the perceptions about ZIKV infection among mothers of children born with microcephaly during the ZIKV epidemic in the Caribbean Colombia, and the barriers and facilitators affecting child health follow up. An exploratory qualitative study, based on Phenomenology and Grounded Theory, was conducted in the Caribbean Colombia. Data were collected through In-Depth Interviews (IDI) from women who delivered a baby with microcephaly during the ZIKV epidemic at Clínica Salud Social, Sincelejo, Sucre District. Themes that emerged during the interviews included experiences from their lives before pregnancy; knowledge about ZIKV; experiences and perceptions when diagnosed; considering a possible termination of pregnancy, and children's clinical follow up. In some cases, women reported having been told they were having a baby with microcephaly, but decided not to terminate the pregnancy; while in other cases, women found out about their newborn's microcephaly condition only at birth. The main barriers encountered by participants during children follow up included the lack of psychosocial and economic support, the stigmatization and abandonment by some partners and relatives, and the frustration of seeing the impaired development of their children.

Conclusions

This study contributed to identifying social, medical, psychological and economic gaps of families with children heavily affected by the ZIKV epidemics. Addressing these gaps is essential to ensure access to quality health care by affected children and their families.

Author summary

As of January 2018, nearly 4000 cases of infants with congenital birth defects associated with Zika virus infection, included microcephaly, had been reported in the Americas. In 2019, we interviewed eleven mothers of children with microcephaly in Colombia, a country heavily affected by the Zika epidemic. Most women were young, living in peri-urban areas, and of low socioeconomic status. They reported knowing that the virus was transmitted by mosquitoes, and how to prevent mosquito bites, yet they were not aware of Zika infection being transmitted by sexual contact. Most women had been tested for Zika during pregnancy but did not receive their laboratory results. Also, they complained that they had not received enough information from healthcare providers. When fetal anomalies were detected prenatally, only few of them were offered the possibility to terminate the pregnancy. Women reported a lack of both economic and psychosocial support to deal with the special health care needs their children required. The results of this study highlight the struggles and challenges of families of children severely affected by the Zika virus, and provide valuable information for health policy making as part of the next epidemic preparedness and response.

Keywords: Views; perceptions, attitudes; Zika; microcephaly; pregnancy; qualitative, grounded theory, Colombia

Introduction

In 2015-17, the epidemic of Zika virus (ZIKV) in the Americas caused a sudden increase in children born with microcephaly, and other neurological manifestations, known as Congenital Zika Syndrome (CZS) [1]. ZIKV infection, especially in the first trimester of pregnancy, was associated with severe

outcomes in fetuses and children [2]. The consequences of CZS not only involve affected children, but their environment, including their community, relatives, and their primary caregivers, usually their mothers, who are more prone to suffer anxiety and depression due to difficulties in providing the specialized care with their children require [3–7]. Mothers of children affected by microcephaly experience significant emotional and psychological distress; as they are stigmatized, abandoned by their partners, relatives and communities [3]. Additionally, it has been reported that their mental health worsens over the years [4,8]. Families were also affected, by changes produced in the routine of the family, which in turn, impacts the couple's relationship, the possibility to work, as sometimes one parent needs to quit the job for child care, leading to economic difficulties in the family [9]. Most affected families are from low socioeconomic status, where ZIKV cases are more prevalent, then, experiencing more restrictions to provide specialized care [3]. Repercussions of having a baby with CZS have been reported to exacerbate social inequalities and poverty [10]. Even when specialized assistance for children was received, women did not have psychosocial support to cope with this stressful situation [11].

According to WHO ZIKV reports, Colombia was the second country with the highest prevalence of ZIKV cases, and CZS babies in the American region, after Brazil [12]. In Colombia, the epidemic led to three to four times as many infants born with microcephaly in 2015-2017 compared to estimates expected in the absence of the virus [13]. Among all ZIKV cases in Colombia, 66% of them were women [14]; and its burden fell on the poorest populations [15]. However, a study performed in Colombia found a lower prevalence of ZIKV cases in poorer areas, but authors hypothesize that could be due to the limited access of healthcare centres in poor areas to report cases, decreased inter-municipal connectivity in those places, and lower access to education, reducing medical attendance and case detection [16]. In 2016, the national Government implemented the Zika Virus Fever Response Plan to strengthen public health surveillance systems, health education, vector control strategies and encouraged research and close monitoring of pregnant women and children's health [14]. On the 7th of January 2016, the Ministry of Health published a notice in which they recommended: "*All couples living in the country not to get pregnant during the epidemic phase, until*

July 2016 [...] and pregnant women who do not live in an area below 2.000 msnm, not to travel to those areas for the high risk of acquiring the infection, until July 2016" [17]. Regarding sexual and reproductive health rights (SRHR), ten years before, in 2006, advocacy efforts contributed to the partial decriminalization of abortion in Colombia in three instances: when pregnancy constitutes a threat to the woman's health or life, if the fetus presents anomalies incompatible with life, or if pregnancy is caused by rape or incest [18,19]. Still, lack of access, makes abortion the fifth leading cause of maternal mortality in the country [18,19]. A qualitative study performed with key informants from the Colombian national and local governments, healthcare providers, community members and affected women, concluded that a multidimensional approach that considers healthcare services, gender issues, and people's environment is crucial for the success of ZIKV campaigns; and that the effects on women's rights are related to inequalities in SRHR (increased risk of sexually transmitted infections by the poorest and most vulnerable women) [14].

Structural gender inequities in Central and South America are well documented, and women often are not in control of their reproductive decisions [15]. Populations most affected by ZIKV often had limited access to reproductive health services, such as antenatal care, contraception counselling and services -including emergency contraception-, safe abortion, and post-abortion care [20,21]. Many countries affected by ZIKV still have restrictive abortion laws, making safe abortion completely illegal or very difficult to access [21]. Furthermore, the epidemic did not change the voluntary abortion landscape in any relevant way. In fact, in Brazil, a law was introduced to increase jail sentences for women seeking abortion "*due to microcephaly or other foetal anomaly*" [22]. In some countries, such as El Salvador and Colombia, authorities recommended sexual abstinence as a preventive measure against ZIKV infection [19,23]. As policies left women solely with the responsibility to avoid pregnancy, in countries where abortion is criminalized, unsafe abortions could have led to rises in maternal morbidity and mortality [3].

ZIKV was the most recent infection disproportionately affecting women in different aspects of health and wellbeing, including their emotional state and mental health. Yet, the social, economic, cultural, and personal consequences of the epidemic remain unknown; and its magnitude represents a

challenge. Furthermore, the effects and ravages that Zika caused in Colombian families, are unknown. The Ebola, Cholera, Zika, and COVID-19 outbreaks raised the need to understand the social pathways of disease transmission and barriers affecting populations at risk [24,25]. The role of Anthropology in emerging outbreaks have raised global awareness for the integration of sociocultural approaches in response to international health crises [24,25]. The present study aimed to explore the views, perceptions, and attitudes, towards ZIKV, and challenges, including barriers and facilitators to medical follow up of children born with microcephaly in the ZIKV epidemic in Caribbean Colombia, faced by their primary caregivers: their mothers.

Methods

Study design

This was an exploratory qualitative study based on a phenomenological approach to understand first-hand experiences of populations under study [26]. Grounded Theory was used as a methodological and analytical approach to inductively create theoretical generalizations that emerge from the data [27].

Study site and population

The study was performed between April and July 2019. Participants were identified from a study that had been conducted in the area of infants with microcephaly from rural and peri-urban areas in Córdoba and Sucre departments, the Caribbean Colombia, born during the ZIKV epidemic at the Clínica Salud Social Hospital, in Sincelejo, Sucre [8,28]. Interviews took place in the departments of Córdoba, Sucre, Bolívar, and Cartagena, according to women's place of residence. Mothers of infants with microcephaly that were born in 2016 were invited to participate in this study. Inclusion criteria for study participants were defined as: having a newborn diagnosed with microcephaly during the ZIKV epidemic and being willing to be interviewed and audio-recorded as part of study procedures.

Data collection

Data collection took place from April to July 2019. Data were collected through In-Depth Interviews (IDI) with primary caregivers (mothers) of children born with microcephaly in 2016. When interviews occurred, children had two or three years of age and in most of the cases, they were present during the interview. The duration of IDIs was around 45-60 minutes. Interviews were carried out at the place of preference of the participants, including health facilities (Clínica Salud Social in Sincelejo), participant´s place of residence or public spaces. All interviews were digitally recorded and notes were taken.

Data analysis

Interviews were transcribed and data were coded using Dedoose® software (SocioCultural Research Consultants, LLC, Manhattan Beach, CA, USA). Consensus on codes and emerging themes were reached in meetings within the investigators´ team. Research began with no pre-existing hypothesis, allowing theories to inductively emerge from the data, following a systematic and circular data collection and analysis, according to Grounded Theory [27]. Theory generation was based on comparative analyses among data collected from different participants, and pre-existing conceptualizations were not used [26,29]. Overlapping themes and subthemes emerging from the participants' narratives are summarized in the Table S1 in the Supporting Information.

Ethics statement

Ethical approval for the study was granted by the Committee of the Universidad de Córdoba, Montería [Reg. No. FMVZ-001-2016] and by the study Clínica Salud Social in Sincelejo Ethics Committee [Reg. No. F-GI-IV-001]. The study was conducted following the Good Clinical Practice Guidelines and under the provisions of the Declaration of Helsinki and local rules and regulations. Participants gave written consent for interviews to take place and be audio-recorded. All names in the transcripts were deleted to guarantee subject anonymity. All names appearing in the manuscript have been created.

Results

Participants' profiles

A total of eleven women whose children were born with microcephaly born during the first semester of 2016 were enrolled in the study. Average age of participants was of 26 years. All female caregivers were the biological mothers of the microcephalic children, except for one who was the grandmother of the child whose biological parents refused to take care of the baby. Table 1 describes the sociodemographic characteristics of study participants.

Table 1. Sociodemographic characteristics of women participating in the study, ZIKV-associated symptoms during pregnancy, and description of microcephaly diagnosis.

**Name***	**Age**	**Education**	**Occupation**	**Relatives living with her**	**Religion**	**SES ******	**ZIKV symptoms** (pregnancy)	**How was maternal ZIKV/microcephalic children being diagnosed?**
**María**	32	Secondary school	Caring of her child	Partner, daughter, and microcephalic child	Christian	Low	Rash, muscular pain and headache (month 3)	All Ultrasound (US) exams were normal, the only concern was that the foetus was underweight. In the last US (month 8), foetal head measurement did not correlate with other anthropometric parameters.
**Nadia**	21	Primary school	Caring of her child	Partner, mother in law, father in law, grandmother in law, and microcephalic child	Christian	Low	Fever, headache and vomiting (early in pregnancy), and does not remember if she presented with any rash.	All US assessments were normal, until the last one, when head circumference was detected to be smaller than expected for gestational age.
**Adelaida**	26	Practical training	Caring of her child	Partner, daughter, and microcephalic child	Christian	Low	Rash and fever (month 3)	She was tested for ZIKV, never received her results. All US were normal, until the last one when "microcephaly" was diagnosed.
**Carla**	30	Not Known	Not Known	Partner, and microcephalic child	Christian	Low	Rash (month 2)	All US exams were normal. At month 7, head circumference smaller for

								gestational age was detected. At month 8, microcephaly was confirmed.
**Guadalupe**	18	University (ongoing)	Student	Mother, two siblings, and microcephalic child	Christian	Low	Rash (before realizing she was pregnant)	First US exam was normal. Patient didn´t attend to second US appointment due to monetary constraints. During third US, measures did not correlate with gestational age, and a C-section was performed.
**Judith**	29	University	Teacher (literates' adults)	Partner, and microcephalic child	Christian	Medium	Rash, fever, and bone pain (not defined)	All US exams were normal. Only concern clinicians had was that the baby had intrauterine growth restriction.
**Georgina**	43	Secondary	Caring of her child	Partner, child, daughter in law, two grandchildren, and the grandchildren with microcephaly (She was the grandmother but primary caregiver)***	Christian	Low	Biological mother presented fever (months 2 or 3)	All US exams were normal. At month 8, microcephaly was confirmed.
**Paola**	23	University	Caring of her child	Father, mother, brother, niece, and microcephalic child	Christian	Low	Rash, fever, and body pain (month 1)	In an US exam (month 6) microcephaly and Dandy–Walker syndrome were diagnosed in the baby.
**Jenny**	20	Primary school	Assistant in a household	Partner, son, microcephalic child, and she's pregnant at the moment of the interview***	Christian	Low	None. But her husband had symptoms compatible with ZIKV when she was in month 5 of pregnancy	All US exams were normal. In the last US exam, microcephaly was diagnosed.
**Consuelo**	22	Primary school	Caring of her child	Partner, mother in law, father in law, and microcephalic child	Not declared	Low	Rash during pregnancy (month not specified)	First US exam was normal. During second US, health staff noticed baby's head was smaller than expected for gestational age. No diagnosis of microcephaly was

								done until baby was born.
**Carmen**	24	Practical training	Nursing assistant**	Her microcephalic child	Christian	Low	Rash and fever (month not specified)	Microcephaly was detected by US. ZIKV screening in child blood samples were then performed.

SES: Socio-economic status; US: Ultrasound. *All names have been made up to guarantee anonymity. **At the moment of the interview, she was on leave to take care of her child who had Dengue virus infection; ***They live in a rural settlement as they were expelled from their home-town by the armed conflict in Colombia. ****According to official government classification in Colombia, low socioeconomic status corresponds to strata 1 and 2; medium is strata 3; and higher is 4-6 [30].

Knowledge of ZIKV and sources of information

Knowledge of ZIKV was very diverse and related to the educational background of the women interviewed. Some women had a very basic knowledge of the virus, with gaps in knowledge in the understanding of Zika being a virus, transmission of the disease and consequences of the disease, expressed by short sentences such as "*Zika is a disease that… is what is happening to my child, is like a bacteria that affects children, is the only thing that I know*" (Jenny, 20 years old), or *"with the fever you have, the rash in the body, with that it is transmitted"* (Nadia, 21 years old). Others had very good knowledge and understanding of the virus and the disease, even including technical terms in their narratives such as *"brain anomalies, microcephaly, eye defects"* as a consequence of infection during pregnancy, or mentioned, "*hemorrhagic Dengue infection*" i.e. *"It's a mild disease [Zika], generally, temporary, as happened to me. It causes pain, and once one is pregnant, it affects very much the brain and well, the normal system of the baby"* (Paola, 23 years old). Knowledge was mentioned to be generally acquired by TV, the Internet, public health campaigns, knowledge from people inside the community and their own experience. While some women changed their behaviour with mass media (i.e. stop watching TV) to protect themselves from seeing images about babies with microcephaly during pregnancy; others expressed the importance of news on TV or the Internet to get informed about Zika, as quoting *"As far as you are, the information does not spread the same*

manner… what helps people more, is always TV” (Judith, 29 years old). Some women expressed the importance of disseminating information about ZIKV to the general public to learn about the disease and how to prevent it, and to stakeholders to help affected families, as illustrated here:

“I think that it [information] *should not remain here like a research study, but it should be information that impacts, that arrives… arrives to other settings, mainly to the areas most at risk, for people to get to know this information. The fact that, not knowing the information that is affecting us, indeed affects us, whether we are infected or not. But the fact of not knowing the information, affects us. In general terms, first, because we are prone to… to live it, second, once we live it, we don’t know how to handle it, thirdly, for all the children, let’s say, it’ll affect them, because if you don’t know how to handle it, you cannot take decisions, right? and the correct support”* (Judith, 29 years old).

Regarding preventive measures, many women felt resignation and rage were expressed as the majority of them were already pregnant when recommendations of “postponing pregnancies” arrived; so, there was not a real option for many of them: *“They started postering in healthcare centers saying ‘These are the symptoms. And then, ‘Please, don’t get pregnant!’, but there were so many pregnant women already [...] Those who are pregnant cannot become “un-pregnant”* (Judith, 29 years old)

Women’s experiences and perceptions regarding their diagnosis of ZIKV infection and or the diagnosis of microcephaly in their babies

Most women declared that they were not ZIKV screened during pregnancy, even though they presented compatible symptoms. Those who were screened never received their results. In both cases, confirmation of maternal ZIKV infection was not possible. They expressed their concerns and feelings of *“rage”* about not having their results, to know the cause of their babies’ congenital anomalies and severe manifestations such as microcephaly, seizures, arthrogryposis, eye and hearing problems. ZIKV diagnosis was based on clinical and epidemiological data. Women described common symptoms of ZIKV that they experienced during pregnancy (i.e. rash, fever, body pain). One participant suggested that women who had ZIKV infection in the third month of gestation (first

trimester), had the most affected children, those suffering the worst consequences, including spastic paralysis, like her baby. One woman also explained that ZIKV infection caused spontaneous abortions in her community as illustrated here: *"In most of the pregnancies... where I live… babies always died in the womb. They did not achieve to progress the pregnancy"* (Paola, 23 years old)

Each woman experienced a very different situation in regards to the diagnosis of anomalies in their children. Some women declared they were told about fetal anomalies during pregnancy *"one was informed about everything that was going to happen, and then one decided whether that was wanted or not"* (Paola, 23 years old). One participant declared that she was told that her baby had *"microcephaly"*, but she did not know what that term meant. She went home, told it to mother-in-law and she looked for the term on the Internet. Then, she felt fear when reading "*Children with microcephaly are those that have a small head, problems and seizures*" (Jenny, 20 years old).

Others knew it once the baby was born *"Zika was a word I learned when [name of the child] was born"* (Judith, 29 years old). One participant explained that she was still on the operating table when the healthcare provider asked her if she had ZIKV as quoted: *"They did me a C-section. They took the baby when the doctor said 'Did you have Zika during pregnancy?' and my world turned upside down"* (María, 32 years old).

Some participants expressed the stigma and discomfort felt caused by some messages on how the idea of children born with CZS is drawn, as expressed in this quote: *"Basically, what was heard by society is that those children needed to be taken out from the womb [...] because they were going to be born like monsters! That was what they said… and sounded horrible [...] or that they would not live more than some months… that they were going to die"* (Adelaida, 27 years old)

Discussions about a possible abortion

Regarding a possible termination of pregnancy, answers and opinions were very diverse; but all women would have appreciated more information about their pregnancies' development,

consequences of maternal ZIKV infection on their children, and to have been approached by healthcare professionals more skillfully to talk about such a sensitive topic. Some women were told that the baby had microcephaly while in the uterus, and could discuss a possible termination of pregnancy. Reasons for refusing it were based on their own decision, their families' decision, or religious beliefs. Sometimes, they felt "*almost forced to terminate the pregnancy*" or judged by healthcare staff and society if they decided to continue their pregnancy.

"What one hears from people [...] Why are you going to have a baby like this? Why?' They are so cruel… After I had her… 'Why did you have her? You're so selfish, you had the opportunity to avoid [your baby] having such a limited life'… and 'what makes you feel happy to have brought a baby who could never walk?' And lots of attacks…" (Paola, 23 years old)

In other cases, women were not given the possibility to terminate their pregnancy. Some women hesitated about the fact that healthcare providers could have known in advance information about the health status of their fetuses, so they could have discussed different options. But most of the time, the diagnosis of anomalies came too late in pregnancy or after delivery. Misinformation and absence of options regarding SRHR were noted in participants interviews:

Interviewee: "At any time, did they tell you that there was a possibility to terminate the pregnancy?"

Consuelo: "They told me it was too late [...] It was 6 months of pregnancy and that was not an option then. [...] We would indeed have interrupted the pregnancy because we were not prepared for this. So, if we would have known it on time… we would have interrupted the pregnancy"

(Consuelo, 22 years old)

One woman explained that, even though she decided to continue her pregnancy, she would support other women and respect their personal decision of having, or not, having the baby. She said that this is a very difficult decision but needs to be addressed by each woman and her family, according to the

circumstances in which they live, as these children would need special care throughout their lives. When prompted about what she would say about a woman whose fetus has been diagnosed with microcephaly, she stated *"Well, first… I would respect her decision, because this is something personal, because if she says she cannot have it, well, in my case, I would say I support her, I would say… I would comment on my situation with the child and so...* (Carmen, 23 years old)

Direct and indirect effects of carrying a child with microcephaly

During the interviews, women expressed the feelings they experienced while being diagnosed with ZIKV, and during childcare. Common feelings and emotions expressed were fear, rage, and sadness. Women talked about fear when they knew they were ZIKV infected, but also for the future, as most of them fear that they, or any other family member, could have another child with microcephaly. Rage was expressed mainly when revealing that they did not receive their ZIKV screening results, and the lack of support they received from local and national authorities. Sadness and suffering are mentioned when talking about their child development, either because he/she is not able to walk, or if able, suffering not to hurt other mothers. One mum mentioned that her child is the only one capable of walking in this group of children, and when the other mothers see her child in the clinic, they start walking. This mum felt pity and worried about them, so she asked the doctor to attend to them first to avoid being in the waiting room with the other families with affected children. Happiness, and gratitude are also mentioned while talking about their children's development, religious beliefs and hope to be heard and supported. Stigma was felt in certain ways throughout the interviews. Some families disappeared, as they did not want to have a relationship with the baby (sometimes, it was thought to be due to avoid future borrowing of money); even biological parents left one of these children with his grandmother. These aspects change behaviours. Some women expressed that during pregnancy they avoided watching TV because they received terrible images of babies with microcephaly. Once they were born, mothers had to change their daily routines to take care of their babies and monitor them all day and night, and they had to learn how to do specific care procedures at home, as almost all of them explained that they are not happy with the quality of the therapies

provided by the health centre. These cross-cutting issues identified within all the themes discussed are detailed in Figure 1.

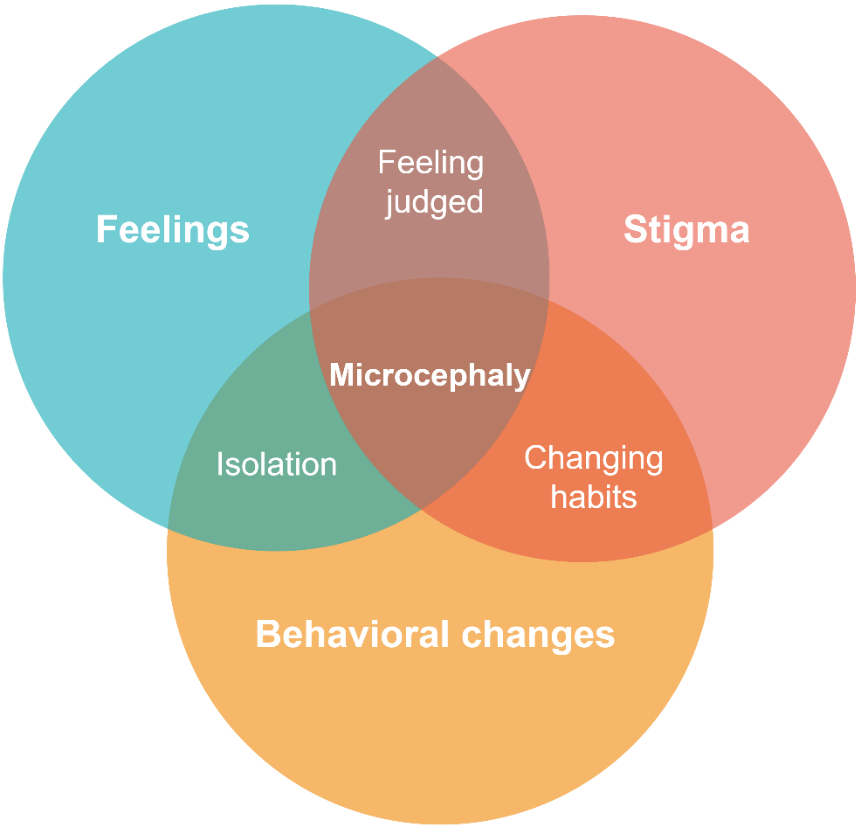

Fig 1. Cross-cutting issues of first-hand experiences of mothers of microcephalic children.

Most women, while talking about the difficulties faced raising a child with special needs, also mentioned how *"lovely and happy"* they and their children are; and that their existence is their main motor in life. But also, a participant declared that they "were not ready" for assuming the challenges of caregiving a child with so many special needs. Some direct and indirect effects of caregiving a child with microcephaly in an endemic area for ZIKV, and a resource-constraint setting are detailed in Table 2.

Table 2. Indirect effects on the families with children with ZIKV-associated microcephaly according to participants

**Area**	**Sub-area**	**Highlights and quotes**

**Individual spheres**	**Motivation**	• Change habits (wake up early and do exercise)<br>• Self-motivation to keep going (great spirit of overcoming)<br>• Start studying Nursing at University<br>*"My therapy is to get up early and exercise [...] because before, I was just crying, I didn't even wake up from bed [...] I said 'I'm going to wake up early every morning' and it's what has helped me the most. Because I go [running] from 4 to 5:30 am"* (María, 32 years old)<br>*"My family was not expecting that I had such a good scor*e [for enrolling at University] *because of the child. But I always had in mind that I had to fight for him, I had to have a good score for him* [microcephalic child]. *Everything that I have proposed to myself now if for him"* (Guadalupe, 18 years old)<br>*"Sometimes I feel alone because it's tough, every day the same routine; one gets bored. But I know that I decided to continue and the main support of [name of his baby] it's me!"* (Carla, undefined age)
	**Religiosity**	• Stronger religious beliefs<br>• Religion/spirituality was a coping mechanism for families to try to explain to themselves why they delivered a child with microcephaly<br>• Religion/spirituality was mentioned as an explanation to refuse a termination of pregnancy as it was believed to be *"God's will"*<br>• Religion/spirituality provided them strength to continue with their lives and take care of the baby<br>• Religion/spirituality makes them believe their children will improve (i.e. walk or talk)<br>"*Sometimes they say to you 'No, no, the girl is not going to walk'. The healthcare professional says one thing and God says another one* [because this child can walk]" (María, 32 years old)<br>"*For a reason God wanted to send him to us*" (Nadia, 21 years old)<br>*"God took the decision that she had to live"* (Judith, 29 years old)
**Social networks**	**Partner**	• More attached now, better relationship<br>• Does not want to take responsibilities on the baby<br>• Abandon them<br>• Started having unhealthy habits (alcohol abuse)<br>• Stopped them from having other children, until the microcephalic child is "self-sufficient"<br>• Stopped them from having other children because nobody assured them that microcephaly was a cause of Zika virus infection, and they are afraid of a congenital disease another child could inherit<br>"*I cannot complain, because I had support from my family, especially from his father [of the baby] because… [she starts crying] there are fathers that abandon their children… [crying] There are fathers that left mothers alone with their [microcephalic] babies, I saw it*" (Carla, undefined age)
	**Relatives**	• Lack of contact with relatives who do not accept the child<br>• Biological parents abandoned the child<br>• Support from their relatives<br>*"Sometimes there is not enough money, then they* [relatives] *always cooperate with us"* (María, 32 years old)
	**Other children within the family (siblings)**	• Worsening of mental health<br>• Does not feel prioritized<br>• Responsibility to take care of the baby when their mum is not at home or need to accompany them to therapies

		• Made her change her mind in her future career, to study physical therapies to attend children<br>*"My older daughter is still really affected* [by having a microcephalic sister]. *I am going to look for a psychologist for her because sometimes she is normal, and others, she's crying, and crying, and crying for* [name of microcephalic child], *because she cannot walk, is not improving…"* (María, 32 years old)<br>*"It's tough for his sister to know that we give him* [microcephalic child] *lots of love, and not to her* [normocephalic sister]*"* (Adelaida, 27 years old)
	**Group of mothers with children with microcephaly**	• They meet with their children and see their progresses<br>• They talk and give advices to each other<br>• They practice sport together<br>*"Just by social media one talk to another, about the child that has something or another thing… but with time everything gets monotony, because they are all the same way, and so, the moment arrives that we don't talk about the babies, we don't know what to say [...] at least, we support each other, what for? 'We can do this, or this [to the baby]' among us… Because there's no support… governmental support, or from other people to help us, just us. If a child has something 'You can solve it with this' or a drug that he takes I say 'Well, this is happening, you can give this [to the baby]'"* (Adelaida, 27 years old)
**Socio-economic context**	**Economic**	Already poor families have decreased their incomes by several reasons:<br>• Not able to study or work outside the home and need to take special attention to the baby 24h a day. One interviewee sells refreshments and sweets at home, as that is the only thing she could do while staying home taking care of the child.<br>• Children need to attend several different medical specialists' different days and on different locations: paediatrics, infectiology, physiatry, among others<br>• Children need to attend neurodevelopmental therapies every day or at least twice a week<br>• Hard decisions are taken by some families: 'to eat' one day or 'to go attend therapy'<br>• Some relatives refused to take care of the microcephalic child. One interviewee declared that her daughter and her son-in-law left their child with her (biological grandmother) and did not provide economic support either.<br>*"In therapy, they only give me 5000 pesos* [to attend the visit]*" [5000 Colombian pesos corresponds to 1.4 USD]"* (Nadia, 21 years old)<br>"*[The health centre] does not support us. They only support the children that need to come from outside the city"* (Carla, undefined age)
	**Psycho-social**	• Some woman received counselling, they talk to psychosocial workers while the children are in therapy<br>• Others, complain about the lack of psychosocial support available for these families<br>• Mental health of the mothers and other family members worsened during these years<br>*"During children's therapies there are psychologists that take some time for us also. They help us talking to express how we feel, we get relieved"* (Georgina, 43 years old)<br>*"One feels alone, sincerely, from the government, from society, and from a lot of people that surround us, sometimes even from family members"* (Adelaida, 27 years old)

Main barriers and facilitators affecting adherence to medical follow up of children with microcephaly

Mothers of children with microcephaly explained the huge range of defects their children present: neurodevelopmental delay, seizures, cerebral palsy etc.; and, that they require specific drugs, vaccines, and therapies, to calm them, and eventually stand up and/or walk. Women explained that they need to attend daily or weekly therapies in specific clinics, and try to do daily therapies at home. While the majority of women complained that those weekly visits are far from the ideal treatment their children needed, they also expressed the barriers they had to overcome to take them to the clinic.

One woman explained that microcephalic children' neurodevelopment highly depends on mothers' support, by all means (economic, educational, familiar…). To note, this family lives in the most socio-economically advanced household compared to the rest of the group; and they are aware that her child is the one who has developed the most (she can walk, attends school, and says a few words), quote here: *"Children's improvement from all this process of therapy highly depends on mothers' support. For example, the fact that my husband supported me from day one is very important for me. He worried because if he had to look for a ticket for going to the city* [where clinical exams are being performed]*, he would do it"* (Judith, 29 years old)

Additionally, one woman explained the great responsibility that is having to raise a baby with so many several anomalies, in a moment when still there are no specific treatments for children exposed to ZIKV while in the uterus, and their development is uncertain: *"We do not have the knowledge to carry out this huge responsibility, that sometimes, we don't know how to handle it"* (Adelaida, 27 years old).

The main barriers and facilitators detected affecting children follow up are described in Table 3.

Table 3. Main barriers and facilitators affecting adherence to medical follow up of microcephalic children

Categories	Barriers	Facilitators
**Personal motivation**	• Not seeing that specialized care received is significantly improving children' neurodevelopment or abilities.	• Realising that the baby' neurodevelopment has improved with specialized care received
**Social**	• Absence of partner/relatives' support.<br>• Difficulties in finding somebody to look for these babies, if mothers need to be outside from home, as they need specialized care.	• Relatives and neighbours sometimes care for children from other families.<br>• Close family/neighbours' relationships facilitate caregiving.
**Logistical**	• Some visits are very far away from their houses (even in different Districts): they lack private transportation, money to pay for public transport, or the baby is too heavy (3 years old) to be carried by their mothers' long kilometres.<br>• Most women lift their children for hours to get to the clinic.<br>• Weather conditions difficult attendance to therapies, especially in the rainy season.	• Referral to the nearest healthcare facility to increase attendance.<br>• Some women have been derived to centres close to their homes. In other cases, there are free buses from the clinic that pick up every mum and baby early morning to get to the therapies.
**Economic**	• Costs associated with specialized drugs and vaccines children need<br>• Costs associated with weekly travel to therapies and monthly/yearly hospital-based exams (food for themselves and their accompaniment, loss of working hours/home chores/schooling for the mothers and their accompaniment…)<br>• Costs associated with impossibility to work outside the home with a dependent child<br>• Lack of a baby trolley to facilitate transportation of the child	• Funds given for travel costs once they are in the clinic, they are more prone to attend those visits.<br>• Monetary compensation to buy essential needs such as diapers, but those require legal demands and a lot of bureaucracy

After the interviews, all women thanked the space for having had the possibility to talk about their processes of pregnancy, their children's health and their family needs; as they did not perceive to

have received psychosocial support or a time to talk to somebody about their experiences and feelings during these tough years: *”There is not any interest here [home country]… to say “let 's help these children, let’s see how mothers are feeling, let’s support them… with company, talk to them to see how they feel”. Thank you for this… keep in mind these children, don’t leave us alone because it’s tough”* (Carla, undefined age)

Discussion

The relevance of this study is vested in the rich in-depth information obtained on several topics raised by women carrying children with microcephaly, who are still facing the consequences of the ZIKV epidemic. In Colombia, the National Healthcare Institute (INS) was in charge of analyzing all ZIKV samples, and updating epidemiological cases; but most results were not given back to patients. In Colombia, ZIKV definition relied on malformations found in fetal ultrasound and maternal clinical and/or epidemiological data. In our study, ZIKV was not confirmed to be the cause of microcephaly, while clinical and epidemiological data suggest it, most mothers were not tested for ZIKV infection, and those tested did not receive their screening results. All children in the study were born in 2016 when the ZIKV epidemic was at its peak, and laboratory resources and capacities were limited in endemic regions. Interviewees will never know if ZIKV was the cause of microcephaly, and this fact has an impact on their daily lives. These women carry the sorrow to remain forgotten by the healthcare system, public health agencies, and local, national, and international institutions. Interviewed women were very young, poor, and with different educational backgrounds. All of them were the biological mothers of children born with microcephaly in Caribbean Colombia in 2016, but one (grandmother but primary caregiver). Some women realized that the baby had microcephaly at delivery, others, late in pregnancy, and were offered a termination of pregnancy, but they refused it for different reasons. These children require special needs, which limits primary caregiver time and economic resources to study, work outside the home, take care of themselves, or other family members. Most women count on their partners and relatives support, and some found shelter in faith, sports, and their motivation to cope with difficulties. Women felt a lack of support and stigmatization (rejection, incomprehension, judgement) in their external social environment, and sometimes even in the most

intimate one, within their families. Stigma is related to those feelings experienced, as women felt judged on many occasions. A woman explained that even though she knew about fetal anomalies, she decided to continue her pregnancy, and her friends judged her and told her she was being "*selfish*" for bringing a child with such special needs. However, a feeling of sorority could be extracted from the interviews, as women talked to the others to get advice (i.e. to apply for specific therapies, monetary compensation etc.), support each other, and see other children's progress. Local, national, and international support to cope with the negative economic, health, and psychosocial effects of the ZIKV pandemic in affected families is almost nonexistent.

Our results show different knowledge levels of ZIKV infection, disease, transmission and prevention. Mostly, women with low levels of education were those having poor knowledge of the virus. Knowledge was also acquired by observations, as one participant correctly mentioned that ZIKV infections in the first trimester caused the most severe outcomes in children. She realized that seeing and asking other mothers of microcephalic children while attending visits in the study clinic. In line with other studies, women were not aware of the sexual transmission of the virus, thus, hampering the use of preventing measures [31–35]. Sexual and reproductive health education should address counselling on ZIKV, preventive methods, diagnosis, and management of the disease [35]. In our study, we noted a lack of SRHR campaigns that should have gone together with recommendations to avoid getting pregnant, along with assured access to contraceptive methods for women among all socio-economic strata. The fact that women declared that messages were prompted to them to avoid getting pregnant, highlighted that ZIKV was conceived as a problem of and for women. Some participants declared their impossibility to do anything as they were already pregnant. It was in line with mass media messages, that the responsibility was put on women's to avoid ZIKV infection, without mentioning the role of men in the sexual transmission of the virus, and men' reproductive capacities and decisions [10,19,36,37]. Responses to public health crisis affecting SRHR, that ask women to postpone their pregnancies, are only feasible if decisions are made by women having complete information, and accessing comprehensive healthcare services within a human rights framework (modern contraception, avoid unplanned pregnancies, legal abortion services etc.) [15].

According to a qualitative study performed with pregnant women in Puerto Rico, authors concluded that public health recommendations to avoid pregnancies must consider how these messages were perceived within specific communities [38]. The prioritization of preventive measures that people could do, such as maintain basic breeding hygiene and removing standing water, is essential for trust-building [38]. These findings are also applicable to our context in which trust in the information received was scarce.

Women in our study who were offered a termination of pregnancy refused to do it based on moral values, either personal or familiar ones and religious beliefs. On the contrary, also some women declared to have preferred to terminate the pregnancy in time if they would know the anomalies their baby presented; but this was a hypothetical situation. Our results are in line with a recent study conducted in Colombia with 21 mothers of children with Zika-associated microcephaly, which found that abortion was not acceptable due to ethical concerns based on religiosity, the idea of maternity, and the patriarchal culture [39]. In our study, some healthcare providers imposed their social-conservative moral values around abortion saying to women that it was "*too late*" to terminate a pregnancy when the woman was still in the second trimester of pregnancy; and the legal regulations allow an interruption of pregnancy. There were several barriers to overcome to obtain a safe abortion in Colombia [18]. While legal at any gestational age, abortion remains highly stigmatized in the country, and only 11% of facilities eligible to offer it, actually do so [18]. Even though abortion is not criminalized in Colombia, social decriminalization did not happen yet. For abortions to become socially accepted, there is a great need for educational work, so that women could be informed about different options to choose from during pregnancy; and have the access to perform it. Efforts are needed to provide sufficient training to healthcare providers to sensitively approach women and not to impose healthcare staff moral values on women's reproductive health decisions. Educational work directed to women and healthcare staff would have beneficial support for women not to be judged about their reproductive decisions. Unfortunately, the ZIKV epidemic did not have an impact on the social acceptance of abortion, neither on policies to improve SRHR in countries where those were most needed. Unsafe abortions still affect the same women for whom access to sexual and

reproductive health services was restricted [15]. Women were forced to face the challenges of giving birth to children with severe neurological malformations, increasing the already existing burden they faced before the epidemic, by their precarious and vulnerable living conditions [15].

According to a study that estimated the incidence of global unintended pregnancies, more than half of all pregnancies in Central and South America are unplanned, due to lack of access to good quality sexual and reproductive health education and services [34]. Differences in women's SRH access are related to intersectional inequalities; including increased risk of sexually transmitted infections, barriers to access quality primary healthcare, lack of adherence to protocols, stigma and discrimination experienced by the poorest and most vulnerable women [14]. ZIKV amplified hierarchical structures and created inequalities. Stigmatization was not only present when women were infected by ZIKV, but they were judged because of their decisions, whether they continued, or not, their pregnancies, as seen in different interviews.

Besides, healthcare professionals need to provide coping strategies and social support for stress reduction, especially for those families living in low-resource settings [8]. Recent epidemics, such as ZIKV, have spread in poor countries, characterized by structural inequalities, high unemployment rates, poor sanitation, lower healthcare awareness, lack of healthcare access, mental health etc., factors that contribute to viral spread and barriers that hinder adherence to treatment [40]. Support for assessment and follow up of mental health is especially important in women pregnant in a context of great uncertainty of health outcomes, and mothers of already affected children [15]. The plan to address the ZIKV epidemic should go beyond health-related interventions or vector control measures and should incorporate actions to address SRHR [14]. The recognition that the effects of the epidemic affect women and men differently is necessary, along with recognition of ZIKV as a sexually transmitted disease, warrantee ZIKV screening diagnosis to pregnant women, access to contraception and safe abortion [15]. An ethnographic study conducted in Colombia with mothers of microcephalic children highlighted the multiple negative changes that occur in the lives of their mothers, due to self-abandonment, mental and physical health outcomes [41].

The cesarean delivery rate has increased globally in recent decades, mainly attributable to a greater number of procedures performed for slow labour progress, breech presentation or repeats cesarean section [42]. A caesarean section is not routinely performed, and according to WHO it should only represent 10-15% of all deliveries [43]. According to our results, all of the women were offered a c-section, as a clear example of the control that the healthcare system and institutions have over women; and the lack of offering several options for women to decide on their pregnancy processes. This malpractice is embedded in the term *"obstetric violence"* and it is widely associated with gender-based violence [44]. Venezuela was the first country in Central and South America to develop legislation around obstetric violence, as the term encompassing diverse concepts such as disrespectful and abusive treatment of women during pregnancy, childbirth and the postpartum period, unconsented and nonmedically indicated care, and negligence during obstetric emergencies [44].

Children born with CZS have a broad range of long-term intellectual, physical and sensory impairments [45], representing a wide-ranging impact on affected children, their families and society as a whole [46]. Our study shows families need to attend weekly visits to psychiatric therapies, and monthly/yearly visits to different healthcare services (ophthalmology, hearing control, infectology, paediatrics etc.), posing additional challenges for already poor families. Some barriers experienced by women are mainly related to lack of economic support to attend those visits, stipends for food for that day outside the home, for somebody to accompany them, loss of productivity/work day etc. Main facilitators for a correct follow up of these children include monetary support for the visits, support from their couple, relatives, and the community.

All these barriers affect directly not only to mother and child but to the family system [47]. Duttine et al. conducted a meta-analysis about the needs of families of children with CZS and found that the caregivers of children with Zika-associated microcephaly experience challenges in mental health, health care access and quality of life [47]. This is in line with the results found in the current study, within the main barriers of families for achieving treatment adherence are the healthcare access and quality of life. Duttine and colleagues found that financial hardships, difficulties with transport and services, and stigma, were other barriers faces by families [47]. Caregivers must pay transports to go

to different specialists, to go to the hospital, to go to school -if children assist to school-; and, in some cases, caregivers must face stigma [47]. In the current study, one mother pointed out that she felt abandoned by the government, society, and even, by the family members. In the study of Romero-Acosta, et al, some mothers in Colombia also felt stigmatized by their own family [8]. This stigma could be perceived as abandonment from family members, above all, extended family.

Sometimes, religion acted as a coping mechanism for women to follow up with their lives, but also healthy habits such as sport, and inner strength; as in line with other studies [8,31]. Support is also needed for concrete actions and material work for caring for these children [15]. Extensive evidence highlights that children with disabilities suffer from different exclusions related to poverty, malnutrition, vulnerability to violence, poor health and school exclusion [46]. Difficulties will grow as adults with disabilities are less likely to be employed and will face again poverty and social exclusion [46].

The main limitations of the study are that the sample size may not be big, although it does not represent a concern because of the richness of the information obtained. The main strengths of the study lie in the feedback and insight provided directly by affected populations; women who faced the challenges of ZIKV during the 2015-2017 epidemics. Qualitative results need to be interpreted with caution, as generalization cannot be performed. The benefits of speaking the same language (interviewees and interviewers) and being a female interviewer might have increased cooperation from participants who seemed comfortable, willing to share their experiences and participate in-depth in the discussions.

Conclusions

The ZIKV epidemic had devastating consequences on women of reproductive age. This study contributes to the understanding of the health inequities that ZIKV infection posed on women living in ZIKV affected areas, particularly among those living in most resource-constrained settings. Mothers of children born with microcephaly faced barriers that hindered them from accessing to the specialized health care required for their children and the adequate psycho-social support for themselves. Most women had high level of knowledge about ZIKV infection and preventive methods, though they were unaware of the sexual transmission of the virus. Their knowledge about ZIKV was acquired primarily through the mass media, the Internet, and the community, while information provided by healthcare providers on ZIKV and its consequences was scarce or inexistent; including consideration of the possibility of pregnancy termination. Addressing social, medical, psychological and economic gaps faced by families with children heavily affected by ZIKV is essential to ensure access to the best possible health care, so that their children may achieve their greatest potential.

Acknowledgments

We are grateful to the women who participated in the study, their families, and children, for their patience and willingness to contribute to research studies, but mostly, their willingness to help other women. We acknowledge support from the Spanish Ministry of Science and Innovation through the “Centro de Excelencia Severo Ochoa 2019-2023” Program (CEX2018-000806-S), and support from the Generalitat de Catalunya through the CERCA Program.

Author Contributions

Conceptualization: Elena Marbán-Castro, María Maixenchs, Azucena Bardají.

Data curation: Elena Marbán-Castro, Anna Marín.

Formal analysis: Elena Marbán-Castro, Cristina Enguita-Fernàndez, Maria Maixenchs, Anna Marín.

Investigation: Elena Marbán-Castro, Cristina Enguita-Fernàndez, Maria Maixenchs, Azucena Bardají.

Methodology: Elena Marbán-Castro, Cristina Enguita-Fernàndez, Maria Maixenchs, Kelly Carolina Romero-Acosta, Salim Mattar, Germán Arrieta, Anna Marín.

Project administration: Elena Marbán-Castro, Kelly Carolina Romero-Acosta, Salim Mattar, Germán Arrieta.

Resources: Elena Marbán-Castro, Cristina Enguita-Fernàndez, Kelly Carolina Romero-Acosta, Salim Mattar, Germán Arrieta, Maria Maixenchs, Azucena Bardají.

Supervision: Cristina Enguita-Fernàndez, Maria Maixenchs, Azucena Bardají.

Validation: Cristina Enguita-Fernàndez, Maria Maixenchs, Azucena Bardají.

Writing – original draft: Elena Marbán-Castro, Cristina Enguita-Fernàndez, Maria Maixenchs, Azucena Bardají.

Writing – review & ed: Elena Marbán-Castro, Cristina Enguita-Fernàndez, Kelly Carolina Romero-Acosta, Salim Mattar, Germán Arrieta, Anna Marín, Clara Menéndez, Maria Maixenchs, Azucena Bardají.

Funding

This study was funded by the Government of Spain under grant agreement number PI16/0123, ISCIII-AES Proyectos de Investigación en Salud, 2016; granted to A.B. Additionally, E. M-C. was supported by a predoctoral fellowship from “la Caixa” Foundation (ID 100010434); fellowship code LCF/BQ/ES17/11600006. E.M-C. received a mobility grant by Universitat de Barcelona for her stay in Colombia. A.B. was supported by the Ministry of Science, Innovation, and Universities, Government of Spain through a Ramon y Cajal Grant (RYC-2013-14512). ISGlobal is a member of the CERCA Programme, Generalitat de Catalunya. The funders had no role in study design, data collection and analysis, decision to publish, or preparation of the manuscript.

Conflicts of Interest: The authors have declared that no competing interests exist.

References

1. França, T.L.B. de; Medeiros, W.R.; Souza, N.L. de; Longo, E.; Pereira, S.A.; França, T.B. de O.; Sousa, K.G. Growth and Development of Children with Microcephaly Associated with Congenital Zika Virus Syndrome in Brazil. *Int. J. Environ. Res. Public Health* **2018**, *15*, doi:10.3390/ijerph15091990.

2. Antoniou, E.; Orovou, E.; Sarella, A.; Iliadou, M.; Rigas, N.; Palaska, E.; Iatrakis, G.; Dagla, M. Zika Virus and the Risk of Developing Microcephaly in Infants: A Systematic Review. *Int. J. Environ. Res. Public Health* **2020**, *17*, doi:10.3390/ijerph17113806.

3. Schwartz, D.A. Clinical Trials and Administration of Zika Virus Vaccine in Pregnant Women: Lessons (that Should Have Been) Learned from Excluding Immunization with the Ebola Vaccine during Pregnancy and Lactation. *Vaccines* **2018**, *6*, doi:10.3390/vaccines6040081.

4. Dos Santos Oliveira, S.J.G.; Dos Reis, C.L.; Cipolotti, R.; Gurgel, R.Q.; Santos, V.S.; Martins-Filho, P.R.S. Anxiety, depression, and quality of life in mothers of newborns with microcephaly and presumed congenital Zika virus infection: a follow-up study during the first year after birth. *Arch. Womens. Ment. Health* 2017, *20*, 473–475.

5. Kuper, H.; Lopes Moreira, M.E.; Barreto de Araújo, T.V.; Valongueiro, S.; Fernandes, S.; Pinto, M.; Lyra, T.M. The association of depression, anxiety, and stress with caring for a child with Congenital Zika Syndrome in Brazil; Results of a cross-sectional study. *PLoS Negl. Trop. Dis.* **2019**, *13*, e0007768, doi:10.1371/journal.pntd.0007768.

6. Kotzky, K.; Allen, J.E.; Robinson, L.R.; Satterfield-Nash, A.; Bertolli, J.; Smith, C.; Ornelas Pereira, I.; Faria E Silva Santelli, A.C.; Peacock, G. Depressive Symptoms and Care Demands Among Primary Caregivers of Young Children with Evidence of Congenital Zika Virus Infection in Brazil. *J. Dev. Behav. Pediatr.* **2019**, *40*, 344–353, doi:10.1097/DBP.0000000000000666.

7. Wiwanitkit, V. Mental Health of Zika Virus-infected Mother and Mother of Newborn with

Microcephaly. *Indian J. Psychol. Med.* **2017**, *39*, 546, doi:10.4103/0253-7176.211750.

8. Romero-Acosta, K.; Marbán-Castro, E.; Arroyo, K.; Arrieta, G.; Mattar, S. Perceptions and Emotional State of Mothers of Children with and without Microcephaly after the Zika Virus Epidemic in Rural Caribbean Colombia. *Behav. Sci. (Basel, Switzerland)* **2020**, *10*, doi:10.3390/bs10100147.

9. Freire, I.M.; Pone, S.M.; Ribeiro, M. da C.; Aibe, M.S.; Pone, M.V. da S.; Moreira, M.E.L.; Dupret, L. Congenital Zika virus syndrome in infants: repercussions for the promotion of families' mental health. *Cad. Saude Publica* **2018**, *34*, e00176217, doi:10.1590/0102-311X00176217.

10. Freitas, P.S.S.; Soares, G.B.; Mocelin, H.J.S.; Lamonato, L.C.X.L.; Sales, C.M.M.; Linde-Arias, A.R.; Bussinger, E.C.A.; Maciel, E.L.N. How do mothers feel? Life with children with congenital Zika syndrome. *Int. J. Gynaecol. Obstet.* **2020**, *148 Suppl*, 20–28, doi:10.1002/ijgo.13044.

11. Santos-Pinto, C.D.B.; de Almeida Soares-Marangoni, D.; Ferrari, F.P.; Ajalla, M.E.A.; Venancio, F.A.; da Rosa, T.S.; de Oliveira, E.F. Health demands and care of children with congenital Zika syndrome and their mothers in a Brazilian state. *BMC Public Health* **2020**, *20*, 762, doi:10.1186/s12889-020-08880-6.

12. Marbán-Castro, E.; Arrieta, G.J.; Martínez, M.J.; González, R.; Bardají, A.; Menéndez, C.; Mattar, S. High Seroprevalence of Antibodies against Arboviruses among Pregnant Women in Rural Caribbean Colombia in the Context of the Zika Virus Epidemic. *Antibodies (Basel, Switzerland)* **2020**, *9*, doi:10.3390/antib9040056.

13. Adamski, A.; Bertolli, J.; Castaneda-Orjuela, C.; Devine, O.J.; Johansson, M.A.; Duarte, M.A.G.; Farr, S.L.; Tinker, S.C.; Reyes, M.M.M.; Tong, V.T.; et al. Estimating the numbers of pregnant women infected with Zika virus and infants with congenital microcephaly in Colombia, 2015-2017. *J. Infect.* **2018**, *76*, 529–535, doi:10.1016/j.jinf.2018.02.010.

14. Forero-Martínez, L.J.; Murad, R.; Calderón-Jaramillo, M.; Rivillas-García, J.C. Zika and women's sexual and reproductive health: Critical first steps to understand the role of gender in the Colombian epidemic. *Int. J. Gynaecol. Obstet.* **2020**, *148 Suppl*, 15–19, doi:10.1002/ijgo.13043.

15. Vélez, A.C.G.; Diniz, S.G. Inequality, Zika epidemics, and the lack of reproductive rights in Latin America. *Reprod. Health Matters* **2016**, *24*, 57–61, doi:10.1016/j.rhm.2016.11.008.

16. Rees, E.E.; Petukhova, T.; Mascarenhas, M.; Pelcat, Y.; Ogden, N.H. Environmental and social determinants of population vulnerability to Zika virus emergence at the local scale. *Parasit. Vectors* **2018**, *11*, 290, doi:10.1186/s13071-018-2867-8.

17. Lineamientos para la gestión de los planes de contingencia para fiebre Zika y atención clínica en embarazadas con infección por ZIKV y pacientes con complicaciones neurológicas y recomendaciones especiales. Actualización de la circular 043 de 2015. . Mini.

18. Rodriguez, M.I.; Mendoza, W.S.; Guerra-Palacio, C.; Guzman, N.A.; Tolosa, J.E. Medical abortion and manual vacuum aspiration for legal abortion protect women's health and reduce costs to the health system: findings from Colombia. *Reprod. Health Matters* **2015**, *22*, 125–133, doi:10.1016/S0968-8080(14)43788-1.

19. Wenham, C.; Arevalo, A.; Coast, E.; Corrêa, S.; Cuellar, K.; Leone, T.; Valongueiro, S. Zika, abortion and health emergencies: a review of contemporary debates. *Global. Health* **2019**, *15*, 49, doi:10.1186/s12992-019-0489-3.

20. Burke, A.; Moreau, C. Family Planning and Zika Virus: The Power of Prevention. *Semin. Reprod. Med.* **2016**, *34*, 305–312, doi:10.1055/s-0036-1592068.

21. Wenham, C.; Abagaro, C.; Arévalo, A.; Coast, E.; Corrêa, S.; Cuéllar, K.; Leone, T.; Valongueiro, S. Analysing the intersection between health emergencies and abortion during Zika in Brazil, El Salvador and Colombia. *Soc. Sci. Med.* **2021**, *270*, 113671, doi:10.1016/j.socscimed.2021.113671.

22. Carabali, M.; Austin, N.; King, N.B.; Kaufman, J.S. The Zika epidemic and abortion in Latin America: a scoping review. *Glob. Heal. Res. policy* **2018**, *3*, 15, doi:10.1186/s41256-018-0069-8.

23. Langer, A.; Caglia, J.M.; Menéndez, C. Sexual and Reproductive Health and Rights in the Time of Zika in Latin America and the Caribbean. *Stud. Fam. Plann.* **2016**, *47*, 179–181, doi:10.1111/j.1728-4465.2016.00058.x.

24. Stellmach, D.; Beshar, I.; Bedford, J.; du Cros, P.; Stringer, B. Anthropology in public health emergencies: what is anthropology good for? *BMJ Glob. Heal.* **2018**, *3*, e000534, doi:10.1136/bmjgh-2017-000534.

25. Kelly, A.H.; Lezaun, J.; Löwy, I.; Matta, G.C.; de Oliveira Nogueira, C.; Rabello, E.T. Uncertainty in times of medical emergency: Knowledge gaps and structural ignorance during the Brazilian Zika crisis. *Soc. Sci. Med.* **2020**, *246*, 112787, doi:10.1016/j.socscimed.2020.112787.

26. Thyer, B.A.; Holosko, M.J. *Overview of Qualitative Research Methods.*; Handb Soc Work Res Methods., 2016;

27. Glaser, B.G.; Strauss, A.L. The Discovery of Grounded Theory: Strategies for Qualitative Research. *Mill Val. CA Sociol. Press.* **1967**.

28. Mattar, S.; Ojeda, C.; Arboleda, J.; Arrieta, G.; Bosch, I.; Botia, I.; Alvis-Guzman, N.; Perez-Yepes, C.; Gerhke, L.; Montero, G. Case report: microcephaly associated with Zika virus infection, Colombia. *BMC Infect. Dis.* **2017**, *17*, 423, doi:10.1186/s12879-017-2522-6.

29. Cartwright, A.; Onwuegbuzie, A.; Manning, L. Using Paired Depth Interviews to Collect Qualitative Data. *Qual. Rep.* **2016**, *21*, 1549–1573.

30. Departamento Nacional de Estadística. Estratificación socioeconómica, 2019. Available: https://www.dane.gov.co/index.php/sistema-estadistico-nacional-sen/69-

espanol/geoestadistica/estratificacion/468-estratificacion-socioeconomica [Accessed 26 Oct 2020].G.

31. Marbán-Castro, E.; Villén-Gonzalvo, A.; Enguita-Fernàndez, C.; Marín-Cos, A.; Menéndez, C.; Maixenchs, M.; Bardají, A. Uncertainties, Fear and Stigma: Perceptions of Zika Virus among Pregnant Women in Spain. *Int. J. Environ. Res. Public Health* **2020**, *17*, doi:10.3390/ijerph17186643.

32. Nelson, E.J.; Luetke, M.C.; Kianersi, S.; Willis, E.; Rosenberg, M. Knowledge and perceptions of Zika virus transmission in the community of Puerto Plata, Dominican Republic. *BMC Infect. Dis.* **2019**, *19*, 339, doi:10.1186/s12879-019-3952-0.

33. Abu-Rish, E.Y.; Elayeh, E.R.; Browning, M.J. Travelers to U.S.: Zika Virus Knowledge, Attitudes, and Determinants of Practices in the Middle East-Insights for Future Awareness Campaigns. *Int. J. Environ. Res. Public Health* **2019**, *16*, doi:10.3390/ijerph16142517.

34. Gurman, T.; Ballard Sara, A.; Villanueva Lorenzo, F.; Luis, D.; Hunter, G.; Maloney, S.; Fujita-Conrads, R.; Leontsini, E. The role of gender in Zika prevention behaviors in the Dominican Republic: Findings and programmatic implications from a qualitative study. *PLoS Negl. Trop. Dis.* **2020**, *14*, e0007994, doi:10.1371/journal.pntd.0007994.

35. Gomez, H.M.; Mejia Arbelaez, C.; Ocampo Cañas, J.A. A qualitative study of the experiences of pregnant women in accessing healthcare services during the Zika virus epidemic in Villavicencio, Colombia, 2015-2016. *Int. J. Gynaecol. Obstet. Off. organ Int. Fed. Gynaecol. Obstet.* **2020**, *148 Suppl*, 29–35, doi:10.1002/ijgo.13045.

36. Linde Arias, A.R.; Tristan-Cheever, E.; Furtado, G.; Siqueira, E. “Too Much to Ask, Too Much to Handle”: Women’s Coping in Times of Zika. *Int. J. Environ. Res. Public Health* **2020**, *17*, doi:10.3390/ijerph17124613.

37. Ribeiro, B.; Hartley, S.; Nerlich, B.; Jaspal, R. Media coverage of the Zika crisis in Brazil: The construction of a “war” frame that masked social and gender inequalities. *Soc. Sci. Med.*

2018, *200*, 137–144, doi:10.1016/j.socscimed.2018.01.023.

38. Horan, H.; Cheyney, M.; Nako, E.; Bovbjerg, M. Maternal stress and the ZIKV epidemic in Puerto Rico. *Crit. Public Health* **2020**, 1–11, doi:10.1080/09581596.2020.1808189.

39. Laza-Vásquez, C.; Rodríguez-Vélez, M.E.; Lasso-Conde, J.; Gea-Sánchez, M.; Briones-Vozmediano, E. [Experience of women with prenatal Zika virus infection who continued gestation in Colombia]. *Gac. Sanit.* **2020**, doi:10.1016/j.gaceta.2020.02.014.

40. Khubchandani, J.; Jordan, T.R.; Yang, Y.T. Ebola, Zika, Corona…What Is Next for Our World? *Int. J. Environ. Res. Public Health* 2020, *17*.

41. Laza-Vásquez, C.; Castaño-Paya, P.A.; Sinisterra-Castro, J.P.; Gea-Sánchez, M.; Briones-Vozmediano, E. [Caring for children with Zika congenital syndrome in Colombia: Consequences for women's lives]. *Aten. primaria* **2021**, *53*, 101972, doi:10.1016/j.aprim.2021.101972.

42. Morton, R.; Burton, A.E.; Kumar, P.; Hyett, J.A.; Phipps, H.; McGeechan, K.; de Vries, B.S. Cesarean delivery: Trend in indications over three decades within a major city hospital network. *Acta Obstet. Gynecol. Scand.* **2020**, *99*, 909–916, doi:10.1111/aogs.13816.

43. WHO. WHO Statement on Caesarean Section Rates. 2015. Available from: https://apps.who.int/iris/bitstream/handle/10665/161442/WHO_RHR_15.02_eng.pdf;jsessionid=A0C7F5A394CD983DE7D3B0002CFE17B7?sequence=1.

44. Williams, C.R.; Jerez, C.; Klein, K.; Correa, M.; Belizán, J.M.; Cormick, G. Obstetric violence: a Latin American legal response to mistreatment during childbirth. *BJOG* **2018**, *125*, 1208–1211, doi:10.1111/1471-0528.15270.

45. Rice, M.E.; Galang, R.R.; Roth, N.M.; Ellington, S.R.; Moore, C.A.; Valencia-Prado, M.; Ellis, E.M.; Tufa, A.J.; Taulung, L.A.; Alfred, J.M.; et al. Vital Signs: Zika-Associated Birth Defects and Neurodevelopmental Abnormalities Possibly Associated with Congenital Zika

Virus Infection - U.S. Territories and Freely Associated States, 2018. *MMWR. Morb. Mortal. Wkly. Rep.* **2018**, *67*, 858–867, doi:10.15585/mmwr.mm6731e1.

46. Lowe, R.; Barcellos, C.; Brasil, P.; Cruz, O.G.; Honório, N.A.; Kuper, H.; Carvalho, M.S. The Zika Virus Epidemic in Brazil: From Discovery to Future Implications. *Int. J. Environ. Res. Public Health* **2018**, *15*, doi:10.3390/ijerph15010096.

47. Duttine, A.; Smythe, T.; Ribiero Calheiro de Sá, M.; Ferrite, S.; Zuurmond, M.; Moreira, M.E.; Collins, A.; Milner, K.; Kuper, H. Congenital Zika Syndrome-Assessing the Need for a Family Support Programme in Brazil. *Int. J. Environ. Res. Public Health* **2020**, *17*, doi:10.3390/ijerph17103559.

Click here to access/download
Supporting Information
4_Supporting information.docx
