# Supplementary material for: “One feels anger to know there is no one to help us!”. Perceptions of mothers of children with Zika virus-associated microcephaly in Caribbean Colombia: A qualitative study
Source: PLoS Negl Trop Dis. 2022 Apr 18;16(4):e0010328. doi: 10.1371/journal.pntd.0010328 (PMC9015150; doi:10.1371/journal.pntd.0010328)
Supplement: Attachment — Submitted filename: Letter with responses_CE_EMC_ce.docx [file pntd.0010328.s003.docx]

Barcelona, 30 August 2021

To the attention of the Editor of the PLOS NTD,

Thank you very much for taking into consideration our manuscript “ *“One feels anger to know there is no one to help us!”: Perceptions of mothers of children with Zika-associated microcephaly in Caribbean Colombia: A qualitative study*".

We are pleased to re-submit a reviewed version of the manuscript. We appreciate the constructive criticisms and we have addressed each of the reviewers´comments and suggestions, as outlined below.

All authors approved the revised manuscript for submission.

Thank you for your consideration.

Kind regards,

Elena Marbán Castro

**Responses to comments from reviewers:**

**Reviewer 1:**

The aims and methods were clearly described. I only have one minor suggestion in the "Abstract" to include the number of mothers interviewed in the methodology description. (it was on the author summary);

Authors’ response: Following your advice, we have included (N=11) in the Methods section of the abstract.

The manuscript is well-written and deals with a very important topic focused on perceptions of mothers of children with Congenital Zika Syndrome.

The results point to a large spectrum based on knowledge about health care, assistance and rights; and some crucial social, subjective, and economic aspects that were affected in their lives.

I have minor comments:

- I liked how you used the tables and figure to describe and summarize the results;

Authors’ response: Thank you very much for your positive feedback.

- Table 2 - include the word "direct" in the description of the table. (Direct and indirect effects...);

Authors’ response: Thank you, that word is included now in the title of the table.

Follow few comments and questions, in case you think they are relevant to your argument in results and discussion:

- The fact of participants live in rural and peri-urban areas with low socio-economic status could be an influence of these mothers' views/perceptions? Could you explore it more in the discussion? Are there studies like this in urban areas in Colombia?

Authors’ response: Indeed, participants socio-economic status influenced the risks of the mothers of having Zika virus infection while pregnant, the lack of possibilities to get diagnosed of ZIKV, the possibilities to get their foetuses diagnosed with microcephaly, and the dayly care provided to these children etc.. We have included a text in the Discussion section to emphasize these ideas: Page 22, lines 431-434: “The low socio-economic status of women in this study could have influenced them for having a higher risk of ZIKV infection, and lower opportunities for being laboratory diagnosed on time, and to have had an ultrasonographic assessment to diagnose fetal microcephaly, as well as the possibilities to provide the special needs these children require.”

To our knowledge, there are not similar studies in urban areas in Colombia to compare results. Though, in the manuscript we have included information from other qualitative studies performed in different areas in Colombia, such as:

Romero-Acosta K, et al. Perceptions and Emotional State of Mothers of Children with and without Microcephaly after the Zika Virus Epidemic in Rural Caribbean Colombia. Behav Sci (Basel). 2020 Sep 25;10(10):147. doi: 10.3390/bs10100147 🡪 This study was performed with mothers of microcephalic children in rural Caribbean Colombia.

Laza-Vásquez C, et al. “It was God’s will”: Continuing pregnancy after perinatal infection by Zika virus. Rev. Latino-Am. Enfermagem. 2020;28:e3310.

Laza-Vásquez C, et al. Qualitative evaluation of a support group for women with children with Zika congenital syndrome in Southern Colombia, Disability and Rehabilitation. 2021. DOI: 10.1080/09638288.2021.1955308

Laza-Vásquez, C. et al. Caring for children with Zika congenital syndrome in Colombia: Consequences for women’s lives]. Aten. primaria 2021, 53, 101972, doi:10.1016/j.aprim.2021.101972. -- both

Laza-Vásquez, C, et al. Experience of women with prenatal Zika virus infection who continued gestation in Colombia]. Gac. Sanit. 2020, doi:10.1016/j.gaceta.2020.02.014.

🡪 In these four studies, authors explored perceptions of women who continued their pregnancy in Huila Department, Colombia. The majority of women were of a low socio-economic status, similar to our study. However, data regarding if populations lived in rural/urban areas was not reported.

- Some studies in Brazil point that groups of mothers with children with microcephaly whether it is online (like WhatsApp groups) or officially organized associations are an interesting way to struggle by rigts, share lived experiences, and have a kind of social support. Have you seen it in your data? I would like to know more about how the mothers share with each other.

Authors’ response: Thanks for this interesting comment. Indeed, we have seen this in our data. Even thought connections among mothers were discussed within the interviews, for instance a mother told us that they have a WhatApp group to communicate among them, and they also have them in other social media such as Facebook; we have not deepened into this topic, as it was not the objective of this study.

- Most of the mothers interviewed had as occupation "caring of her child". Was it a given need by had a child with CZS? To have a child with a CZS has changed their trajectories and project of lives?

Authors’ response: Thank you for this reflection. Indeed, what was extracted from most interviews is that having a child with CZS changed their trajectories to take care of them, but also one woman started to study Nursery at the University to provide her child, and other children better healthcare.

Thanks to your comment, we have included more information in the Discussion section. In page 21, lines 407-409, under the discussion section, this sentence has been added: “Having a child with microcephaly changed women’ trajectories, as most of them reported to be just caregivers due to difficulties in providing special needs and working outside home.”

We would like to thank Reviewer 1 for his/her comments and suggestions.

**Reviewer 2:**

Dear Authors,

I started reading with great interest your article on a topical and important problem that arose after the end of the Zika pandemic, but later I found out that some important corrections need to be made to highlight the value of the article and I present them in detail below.

INTRODUCTION

•	In this section is important to describe in more detail the CZS, the comorbid disorders, as well as its serious effects on the neurodevelopment of infants and children.

Authors’ response: Thank you for this comment, as the spectrum of CZS has been previously explained in several articles, we did not include in the first manuscript much information on it. But, we have included in the section of Introduction, pages 3-4, lines 84-88: “In 2015-17, the epidemic of Zika virus (ZIKV) in the Americas caused a sudden increase in children born with microcephaly, and other neurological manifestations associated with severe brain lesions such as brain disruption, microcalcification, eye damage, hypertonia and arthrogryposis [1,2]. This spectrum of signs and symptoms was reported under the term Congenital Zika Syndrome (CZS) [1,2].”

•	Furthermore, if you have access, it would be great to mention the average monthly income in Colombia, the monthly expenses of a family with a child with CZS and how much the state compensates these families could also be included in a short paragraph.

•	Also, it should be mentioned the percentage of people who are uninsured or living below the poverty line.

•	The insurance system in Colombia:

Authors’ response: Thank you for raising this interesting topic. The reported average monthly income in Colombia is 441 USD.

We have found an article estimating the costs of treatment of a baby born with microcephaly in Brazil (da Silva AL, et al. 2019), but none from Colombia. Thought it might vary for the case of Colombia, it could be an approximate estimate. The estimated annual cost of a child with microcephaly is 5,937 USD.

Regarding the insurance system in Colombia, there exists an employee-based mandatory insurance system, the General System of Health and Social Security (Sistema General de Seguridad Social en Salud; SGSSS). Those people who wish to have additional services may subscribe to the ‘Complementary Plan’ to the ‘Health Promotion Entities’ (Entidad Promotora de Salud; EPS). Poor and unemployed populations are enrolled in the ‘Subsidized Regimen’ (Régimen Subsidiado) paid by the government (Gómez, O.L.; Carrasquilla, G. Factors associated with unjustified Cesarean section in four hospitals in Cali, Colombia. Int. J. Qual. Heal. care J. Int. Soc. Qual. Heal. Care 1999, 11, 385–389, doi:10.1093/intqhc/11.5.385.).

According to the World Bank Group (<https://databank.worldbank.org/data/download/poverty/987B9C90-CB9F-4D93-AE8C-750588BF00QA/SM2020/Global_POVEQ_COL.pdf>) , in Colombia, 13.1 million people live under the national poverty line (27% rate).

We have included a short paragraph in the Introduction section to highlight these ideas, pages 4-5, lines 111-116: “The annual cost of treating a child with Zika-associated microcephaly has been estimated in Brazil to be 5,937 USD, a cost that might be life-long [18]. However, the average monthly income in Colombia is 441 USD, and the official poverty rate was 27% in 2018 [19]. The insurance system for poor and unemployed populations is financed by the government by a ‘Subsidized Regimen’ [20], but the costs to cover the specialized care microcephalic children require are unknown.”

METHODS

•	In the CZS, the spectrum of disability varies depending on the severity of microcephaly. Why was your sample not grouped according to their degree of disability?

Authors’ response: Thank you for this relevant question. This study is a qualitative study where we deeply explored maternal perceptions and views of carrying a child with microcephaly born in the ZIKV epidemic. Not all of them had a confirmatory ZIKV maternal analysis, or a diagnosis of CZS. This study explored the perceptions of women who had a child with microcephaly, this is why we did not included clinical information of the children, nor we grouped them based on their disability.

The results presented clearly and anatically.

Authors’ response: Thank you for this comment.

The tables have sufficient quality.

Authors’ response: Thank you for this comment.

Please explain ''What do you define as “low Socio-economic status?''

Authors’ response: Thank you for your positive comments. The socio-economic status was defined according to official government classification in Colombia, low socioeconomic status corresponds to strata 1 and 2; medium is strata 3; and higher is 4-6. Referenced here: Departamento Nacional de Estadística. Estratificación socioeconómica, 2019. Available: https://www.dane.gov.co/index.php/sistema-estadistico-nacional-sen/69-espanol/geoestadistica/estratificacion/468-estratificacion-socioeconomica [Accessed 26 Oct 2020].

DISCUSION:

•	You refer the cesarean section as a routine procedure in cases with microcephaly. Can you tell if you have access, the rates of cesarean sections in Colombia?

Authors’ response: This might have been a misunderstanding. Having a child with microcephaly was not a routine procedure per protocol; but we noticed in our study that it was a common practice. We have now included a reference of the increase use of uncesarry caesarean sections in Colombia; and a sentence in the Discussion section, page 25, lines 501-503: “However, studies performed in the USA, Italy and Colombia have reported an increase in unjustified Cesarean procedures in private clinics with insured patients, a worrying concern due to the high costs and morbidity associated [20].”

•	The syndrome has a significant psychosocial impact on families, especially mothers and consequently health care providers. Can you report similar studies from all the affected areas?

Authors’ response: The psychosocial impact on families of having a child with microcephaly and/or CZS have been addressed in the following studies which were referenced in the manuscript. However, there are not studies from all affected areas, as ZIKV spread to almost all countries in the American continent, and many other continents.

Most of these studies were conducted in Brazil and Colombia, the two countries with the highest prevalences of ZIKV infections.

Freitas, P.S.S.; Soares, G.B.; Mocelin, H.J.S.; Lamonato, L.C.X.L.; Sales, C.M.M.; Linde-Arias, A.R.; Bussinger, E.C.A.; Maciel, E.L.N. How do mothers feel? Life with children with congenital Zika syndrome. Int. J. Gynaecol. Obstet. 2020, 148 Suppl, 20–28, doi:10.1002/ijgo.13044.

Kotzky, K.; Allen, J.E.; Robinson, L.R.; Satterfield-Nash, A.; Bertolli, J.; Smith, C.; Ornelas Pereira, I.; Faria E Silva Santelli, A.C.; Peacock, G. Depressive Symptoms and Care Demands Among Primary Caregivers of Young Children with Evidence of Congenital Zika Virus Infection in Brazil. J. Dev. Behav. Pediatr. 2019, 40, 344–353, doi:10.1097/DBP.0000000000000666.

Romero-Acosta K, Marbán-Castro E, Arroyo K, Arrieta G, Mattar S. Perceptions and Emotional State of Mothers of Children with and without Microcephaly after the Zika Virus Epidemic in Rural Caribbean Colombia. Behav Sci (Basel). 2020 Sep 25;10(10):147. doi: 10.3390/bs10100147. PMID: 32992703; PMCID: PMC7599807.

Kuper H, Lopes Moreira ME, Barreto de Araújo TV, Valongueiro S, Fernandes S, Pinto M, Lyra TM. The association of depression, anxiety, and stress with caring for a child with Congenital Zika Syndrome in Brazil; Results of a cross-sectional study. PLoS Negl Trop Dis. 2019 Sep 30;13(9):e0007768. doi: 10.1371/journal.pntd.0007768. PMID: 31568478; PMCID: PMC6786834.

Dos Santos Oliveira, S.J.G.; Dos Reis, C.L.; Cipolotti, R.; Gurgel, R.Q.; Santos, V.S.; Martins-Filho, P.R.S. Anxiety, depression, and quality of life in mothers of newborns with microcephaly and presumed congenital Zika virus infection: a follow-up study during the first year after birth. Arch. Womens. Ment. Health 2017, 20, 473–475.

Wiwanitkit, V. Mental Health of Zika Virus-infected Mother and Mother of Newborn with Microcephaly. Indian J. Psychol. Med. 2017, 39, 546, doi:10.4103/0253-7176.211750.

Freire, I.M.; Pone, S.M.; Ribeiro, M. da C.; Aibe, M.S.; Pone, M.V. da S.; Moreira, M.E.L.; Dupret, L. Congenital Zika virus syndrome in infants: repercussions for the promotion of families’ mental health. Cad. Saude Publica 2018, 34, e00176217, doi:10.1590/0102-311X00176217.

Santos-Pinto, C.D.B.; de Almeida Soares-Marangoni, D.; Ferrari, F.P.; Ajalla, M.E.A.; Venancio, F.A.; da Rosa, T.S.; de Oliveira, E.F. Health demands and care of children with congenital Zika syndrome and their mothers in a Brazilian state. BMC Public Health 2020, 20, 762, doi:10.1186/s12889-020-08880-6.

Laza-Vásquez, C.; Rodríguez-Vélez, M.E.; Lasso-Conde, J.; Gea-Sánchez, M.; Briones-Vozmediano, E. [Experience of women with prenatal Zika virus infection who continued gestation in Colombia]. Gac. Sanit. 2020, doi:10.1016/j.gaceta.2020.02.014.

CONCLUSIONS:

•	At this point it is important to emphasize the need to raise awareness of relevant stakeholders, not only locally but also globally. This article highlights not only a significant public health problem and the inequality in access to health services, it presents significant gender inequality issues through presents significant problems of gender inequality through the violation of women's rights and their self-disposition.

Authors’ response: Thank you very much for this powerful message. We have emphasized this in the Conclusions section, page 27, lines 573-576, where it can be read: “It is of high importance to raise awareness of relevant stakeholders locally and globally to respond to all the public health problems including inequalities in access to the healthcare system, gender disparities, and violation of women’s sexual and reproductive rights.”

**Reviewer 4:**

These were described adequately - although there was little description of how participants were selected and recruited. There is also limited discussion of the positionality of the researchers.

Authors’ response: Thank you for this suggestion. We have included more information in the Methods section about selection and recruitment of participants. Page 6, lines 157-165 can read: “Participants were identified from a study that was been conducted in rural and peri-urban areas in Córdoba and Sucre departments, Caribbean Colombia. That study was following up microcephalic children who were born at the Clínica Salud Social Hospital in Sincelejo (Sucre) during the first semester of 2016, during the ZIKV epidemic in Caribbean Colombia [8,28]. Mothers of these children, who were being clinically followed up, were contacted by phone call to be invited to participate in the current study. Inclusion criteria for study participants were defined as: having a newborn diagnosed with microcephaly during the ZIKV epidemic and being willing to be interviewed and audio-recorded as part of study procedures.”

Regarding researchers’ positionality, we would like to thank the reviewer for highlighting this issue. Certainly, it is essential to be aware that the position of the researcher influences how the data is collected and analysed. We have not omitted this reflexive approach while analysing our data. While we agree with the reviewer, it is true that we did not mention it in the first version of the manuscript. To provide a clarification to the reviewer, the main researcher in this study, is a white young woman from Spain, middle-class, well-educated, and financially secure. For this study, a feminist approach has been used in order to deep in the understanding of recently pregnant women who had a child with microcephaly, and are their main caregivers. We think that these perpective may have influenced the area of the study, and even the identification of codes and themes, undertaking a feminist interpretation of Grounded Theory; hence being sensitive and giving special attention to issues related to oppression and gender inequality. For the sake of rigourosity, and to ensure an ethical research, we have included the following paragraph in the limitations section (page 26, lines 545-551): “Regarding researchers’ positionality, the interviewee was a Spanish while young woman, thus participants’ responses might have been affected by a social desirability bias, inherent to unconscious biases such as interviewers’ nationality, employment, economic status, etc. Additionally, a feminist interpretation of Grounded Theory was used by the researcher to give special attention to issues related to oppression and gender inequality, such as daily caregiving of these children, impact in their lives, discussions about a possible termination of pregnancy, or other issues related to sexual and reproductive health.”

There are many linguistic errors, some of which I have highlighted.  The manuscript should be careful read through and edited to address these.
The results are presented as themes and described in text, tables and a diagram. It is adequate

Authors’ response: Thank you very much for highlighting the errors. We have worked on the manuscript, and it has been reviewed by an English native speaker colleage to improve it. Thanks for the suggestion.

Overall the conclusions were supported well by the data presented and the limitations given.  There was perhaps more that could have been done to explore the positionality of the researchers and how this might have influenced the interviews and the analysis of the data.

Authors’ response: Thank you very for raising this topic. While it is very important, we would not want to dedicate much space for this issue, as the main focus of the paper is to give voice to silenced women, and to have space for showing results and discussing them. Adding much information on the positionality of researchers might deviate the focus of attention. However some lines have been added in the manuscript in the limitation paragraph (page 26, lines 545-551): “Regarding researchers’ positionality, the interviewer was a Spanish white young woman, thus participants’ responses might have been influenced by the interviewer characteristics and status (nationality, employment, economic status, etc). Additionally, a feminist interpretation of Grounded Theory was used by the researcher to give special attention to issues related to oppression and gender inequality, such as daily caregiving of these children, impact in their lives, discussions about a possible termination of pregnancy, or other issues related to sexual and reproductive health. The way these positionings might have framed social desirability in participants’ responses should not be disregarded.”

A conclusion that the Zika virus has differential affect on men and women is hard to support without having included men in the research.

Authors’ response: Thank you for this comment.

In the manuscript we cited an article to emphasize that: “The recognition that the effects of the epidemic affect women and men differently is necessary, along with recognition of ZIKV as a sexually transmitted disease, warrantee ZIKV screening diagnosis to pregnant women, access to contraception and safe abortion.”; and also that “The ZIKV epidemic had devastating consequences on women of reproductive age.”. The recognition of the differential effects that the ZIKV epidemic posed on women, who were pregnant during the times of a new epidemic, is a fact, as well as that the majority of primary caregivers of children affected by microcephaly, are their mothers.

As stated in the manuscript, a phenomenology approach was used. In phenomenology, relity is comprehend through embodied experiences of those individuals who experienced the phenomenom under investigation ‘give birth of a microcephalic child or being their primary caregiver’, was used. This implied that all participants in this specific study, were women. Although diverse samples might provide a broader range from which to distill the essence of the phenomenon, data from few individuals who lived it, can provide detailed experiences to uncover core elements (from 1 to 10 individuals) [Starks H. Choose Your Method: A Comparison of Phenomenology, Discourse Analysis, and Grounded Theory. 2013. Qualitative Health Research.]. For that reason, this specific study was not conceived to include men’ perspectives. However, having analysed and explored all the areas of interest discussed in this study, researchers understand that further studies are needed to explore masculine perspectives to enrich conclusions obtained in our study; and it has been added in the manuscript as follows (page 27 lines 557-562): “Further studies are needed to understand other mothers of children born with microcephaly during the ZIKV epidemic, as well as studies including perspectives of other caregivers rather than their mothers, fathers, and other members of the families. The perspectives of men regarding childcare, sexual and reproductive health decisions, discussions about a possible termination of pregnancy, their role within the family and community, are interesting topics of discussion to be explored in further research.”

This is an important study exploring the views and experiences of mothers or primary caregivers caring for an infant that has Sika associated microcephaly in Caribbean Colombia.  The weakness of the paper that need to be addressed are: improved description of their methods including recruitment, translation in transcription or at report writing and the positionality of the researchers.  There are numerous errors, some of which I have highlighted on the pdf of the manuscript which need to be corrected.

Authors’ response: Thank you very much for highlighting the errors. We have worked on the manuscript, and it has been reviewed by an English native speaker colleage to improve it. Thanks for the suggestion.

We would like to thank all reviewers and editors for their valuable time to review this manuscript. In this new version, all suggestions have been considered to meet publication’s expectations.

Kind regards.
