# Supplementary material for: “One feels anger to know there is no one to help us!”. Perceptions of mothers of children with Zika virus-associated microcephaly in Caribbean Colombia: A qualitative study
Source: PLoS Negl Trop Dis. 2022 Apr 18;16(4):e0010328. doi: 10.1371/journal.pntd.0010328 (PMC9015150; doi:10.1371/journal.pntd.0010328)
Supplement: Attachment — Submitted filename: Dear Editor.docx [file pntd.0010328.s005.docx]

Dear Editor,

Unfortunately, the authors did not revise the manuscript according to our suggestions.

INTRODUCTION
•     In this section is important to describe in more detail the CZS, the comorbid disorders, as well as its serious effects on the neurodevelopment of infants and children.
•     Furthermore, if you have access, it would be great to mention the average monthly income in Colombia, the monthly expenses of a family with a child with CZS and how much the state compensates these families could also be included in a short paragraph.
•     Also, it should be mentioned the percentage of people who are uninsured or living below the poverty line.
•     The insurance system in Colombia
METHODS
•     In the CZS, the spectrum of disability varies depending on the severity of microcephaly. Why was your sample not grouped according to their degree of disability?

The results  presented clearly and anatically
The tables have sufficient quality
Please explain ''What do you define as “low Socio-economic status?''

DISCUSION
•     You refer the cesarean section as a routine procedure in cases with microcephaly. Can you tell if you have access, the rates of cesarean sections in Colombia?
•     The syndrome has a significant psychosocial impact on families, especially mothers and consequently health care providers. Can you report similar studies from all the affected areas?
CONCLUSIONS
•     At this point it is important to emphasize the need to raise awareness of relevant stakeholders, not only locally but also globally. This article highlights not only a significant public health problem and the inequality in access to health services, it presents significant gender inequality issues through presents significant problems of gender inequality through the violation of women's rights and their self-disposition.

Dear Editor
Thank you for the invitatation to review this manuscript. I suggest minor revisions

Kind regards

INTRODUCTION
•     In this section is important to describe in more detail the CZS, the comorbid disorders, as well as its serious effects on the neurodevelopment of infants and children.
•     Furthermore, if you have access, it would be great to mention the average monthly income in Colombia, the monthly expenses of a family with a child with CZS and how much the state compensates these families could also be included in a short paragraph.
•     Also, it should be mentioned the percentage of people who are uninsured or living below the poverty line.
•     The insurance system in Colombia
METHODS
•     In the CZS, the spectrum of disability varies depending on the severity of microcephaly.

Why was your sample not grouped according to their degree of disability?
RESULTS
•     What do you define as “low Socio-economic status”?

DISCUSION
•     You refer the cesarean section as a routine procedure in cases with microcephaly. Can you tell if you have access, the rates of cesarean sections in Colombia?
•     The syndrome has a significant psychosocial impact on families, especially mothers and consequently health care providers. Can you report similar studies from all the affected areas?
CONCLUSIONS
•     At this point it is important to emphasize the need to raise awareness of relevant stakeholders, not only locally but also globally. This article highlights not only a significant public health problem and the inequality in access to health services, it presents significant gender inequality issues through presents significant problems of gender inequality through the violation of women's rights and their self-disposition
