# Supplementary material for: “One feels anger to know there is no one to help us!”. Perceptions of mothers of children with Zika virus-associated microcephaly in Caribbean Colombia: A qualitative study
Source: PLoS Negl Trop Dis. 2022 Apr 18;16(4):e0010328. doi: 10.1371/journal.pntd.0010328 (PMC9015150; doi:10.1371/journal.pntd.0010328)
Supplement: Attachment — Submitted filename: Response_reviewers_ce_mm_emc.docx [file pntd.0010328.s007.docx]

Dear editors and reviewers,

Thank you very much for your time to review our manuscript.

Here it is a detailed list of our responses to reviewers’ comments, including the description of the changes made in the manuscript.

**INTRODUCTION**

**Reviewer #1: The aims and methods were clearly described and more details about participants' recruitment were pointed.**

Authors’ response: Thanks for acknowledging it.

**Reviewer #2:**

**•	In this section is important to describe in more detail the CZS, the comorbid disorders, as well as its serious effects on the neurodevelopment of infants and children.**

Authors’ response: Thought, the aim of the study was not to characterize the spectrum of anomalies within this syndrome, we have included some additional information in the Introduction section (pages 3-4, lines 88-93): “In 2015-17, the epidemic of Zika virus (ZIKV) in the Americas caused a sudden increase in children born with microcephaly, and other neurological manifestations associated with severe brain lesions such as brain disruption, microcalcification, eye damage, hypertonia, and arthrogryposis [1,2]. This spectrum of signs and symptoms was, described under the term Congenital Zika Syndrome (CZS) [1,2]. CZS includes a diverse pattern of structural brain anomalies and functional disabilities in affected children, secondary to central nervous system damage [3]. Microcephaly is its most common sign, observed in 80% of CZS cases [3] .”

**•	Furthermore, if you have access, it would be great to mention the average monthly income in Colombia, the monthly expenses of a family with a child with CZS and how much the state compensates these families could also be included in a short paragraph.**

**•	Also, it should be mentioned the percentage of people who are uninsured or living below the poverty line.**

**•	The insurance system in Colombia**

Authors’ response:

All these three points were addressed in page 5, lines 117-128: “The real costs to cover the specialized care that microcephalic children require are unknown, and might be life-long. Though it is very difficult to estimate the annual cost of having a ZIKV-associated microcephalic child, a study in Brazil estimated that the healthcare treatment was 5,937 USD per child, per year [19]. ZIKV infection was most prevalent in young women that lived in the outskirts of the cities, and in low-socioeconomic statuses [20]. In Colombia, the average monthly income is 441$, and the poverty rate (population living on less than 5.5$/day) for 2018 was 27% [21]. According to official government classification in Colombia, districts are rated from 1 to 6 for its affluence. Populations living in districts of Status 1 and 2 are considered poor, and are eligible for an insurance financed by the government, entitled ‘Subsidized health Regimen’ (régimen subsidiado) [22,23]. However, being affiliated to this regimen has been associated with poorer health outcomes, due to social determinants of these populations, and barriers in access to healthcare services [24].”

Direct and indirect costs of caring for these children are very difficult to be estimated. This cost has been ranged among 1.300 and 4.800 million dollars; and it might be an underestimation, due to the difficulties in its evaluation. According to one of the few studies that measured the costs of ZIKV-associated microcephaly (UNFPA, The International Red Cross and Red Crescent Societies. A socio-economic impact assessment of the zika virus in Latin America and the Caribbean. 2017); the total estimate cost of microcephalic cumulative cases in the region ranges from 3.000 to 28.900 million dollars; with variations from countries from 800.000 dollars to 1 million dollar.

**METHODS**

**Reviewer #2: In the CZS, the spectrum of disability varies depending on the severity of microcephaly. Why was your sample not grouped according to their degree of disability?**

Authors’ response:

Thank you for giving us the opportunity to discuss it.

First, the impact of the spectrum of disability in women’s experiences would respond to a hypothesis that was not designed in our study. Our methodological approach was grounded theory. We conducted an exploratory study that did not respond to any specific hypothesis. The present study aimed to explore the views, perceptions, and attitudes, towards ZIKV, and challenges, including barriers and facilitators to medical follow-up of children born with microcephaly in the ZIKV epidemic in Caribbean Colombia, faced by their primary caregivers: their mothers. Our study highlights multiple factors involved women’s experiences, that go beyond the spectrum of disability in their children.

Secondly, the spectrum of disability was not a variable collected for this study. Most women never received laboratory results confirming their ZIKV infection, thus infants ZIKV infection was not laboratory confirmed, neither CZS. CZS, as in most studies conducted in Latin American countries, was diagnosed based on clinical and epidemiological data reported by healthcare professionals. However, in this article, to be more prudent, we only describe children as being “microcephalic” and did not focus on characterization of the spectrum of disability of each child. All of them had a life-long disability, highly-potential to be associated with ZIKV infection during pregnancy. Its effects on family life, especially their mothers, was the aim of this specific study.

Lastly, as this is an exploratory study, it is difficult to identify beforehand the characteristics that the sampled population should have. We selected relevant participants for the specific topic of the study, already described in the manuscript. Collection of data on the degree of disability was not coherent to have occurred based on our objectives. This type of sampling is appropriate for exploratory studies in unfamiliar areas, since it might be difficult to identify the groups and characteristics that needed to be included in advance (Ritchie J; Lewis J. Qualitative Research Practice: A Guide for Social Science Students and Researchers.2003. SAGE Publications Ltd).

This study is a qualitative study based on women’s experiences and perceptions of ZIKV infection during pregnancy, and on carrying a microcephalic child. The aim of the study was not to compare responses among population groups, but to deep into information provided by specific individuals. We contacted all women who gave birth to a microcephalic child in 2016 in the study hospital (Clínica Salud Social in Sincelejo). Only one woman did not accept to be interviewed. As the population of study was very specific, it did not allow us to disaggregate the groups in child’ neurodevelopment, social status etc. So, interviewing all but one woman who had a child born with microcephaly during the ZIKV epidemic in Sincelejo, who still continued clinical follow-up; we are confident on the representativeness of our data for the local context. Still, we understand that this article adds relevant data on this specific population group, and that for drawing less biased conclusions, it needs to be added to the scarce literature on the topic.

**Reviewer #4: This study has used qualitative methods to explore the experiences of women who have had a child affected by the Zika virus. The design is appropriate and the authors have highlighted important findings. The study authors did not seek to get a sample that represented different socioeconomic groups or ensure that their sample was representative in terms of certain factors. This would have strengthened the work.**

Authors’ response:

Thank you very much for highlighting it. We understand the importance of representation in sampling, especially in quantitative studies. However, this is a qualitative study performed with caregivers, mostly biological mothers, of children born with microcephaly during the ZIKV epidemic in Caribbean Colombia in 2016. The aim of the study was not to generalize about experiences in motherhood having ZIKV affected children; but to explore perceptions and experiences among a very specific population group.

This is an exploratory study, it is difficult to identify beforehand the characteristics that the sampled population should have. We selected relevant participants for the specific topic of the study, already described in the manuscript. Disaggregation of data by socio-economic status was not in line with our objectives. This type of sampling is appropriate for exploratory studies in unfamiliar areas, since it might be difficult to identify the groups and characteristics that needed to be included in advance (Ritchie J; Lewis J. Qualitative Research Practice: A Guide for Social Science Students and Researchers.2003. SAGE Publications Ltd).

We understand that there are countless factors (social support, socioeconomic group, child degree of disability, relation with the father etc.) that could differentiate all of these women. However, we contacted all women who gave birth to a microcephalic child in 2016 in the study hospital (Clínica Salud Social in Sincelejo). Only one woman did not accept to be interviewed. As the population of study was so specific, it did not allow us to disaggregate the groups socioeconomic status, etc. So, interviewing all but one women who had a child born with microcephaly during the ZIKV epidemic in Sincelejo, who still continued clinical followed-up; we are confident on the representativeness of our data for the local context. Still, we understand that this article adds relevant data on this specific population group, and that for drawing less biased conclusions, it needs to be added to the scarce literature on this specific population group.

**RESULTS**

**Reviewer #4: There are some important findings, particularly the way the Zika infections exacerbated inequalities, the poor access to family planning services, the lack of access to information, the stigma experienced by women. The findings give valuable information for ways that future outbreaks can be managed and what information needs to be disseminated to vulnerable populations and the nature of the support needed for women in these communities.**

Authors’ response: Thank you for acknowledging that.

**Reviewer #1: The manuscript is well-written and deals with a very important topic focused on perceptions of mothers of children with Congenital Zika Syndrome.**

**The results point to a large spectrum based on knowledge about health care, assistance, gender and rights; and some crucial social, subjective, and economic aspects that were affected in their lives.**

**The tables and figure describe and summarize the results in a good way.**

Authors’ response: Thanks for the nice words. Very much appreciated.

**Reviewer #2: The results presented clearly and anatically**

**The tables have sufficient quality**

**Please explain ''What do you define as “low Socio-economic status?''**

Authors’ response:

Thank you for your feedback. We had that information in Table 1, but we agreed that it needed to appear before. We have added the following sentence in page 5, lines 123-128: “According to official government classification in Colombia, districts are rated from 1 to 6 for its affluence. Populations living in districts of Status 1 and 2 are considered poor, and are eligible for an insurance financed by the government, entitled ‘Subsidized health Regimen’ (régimen subsidiado) [22,23]. However, being affiliated to this regimen has been associated with poorer health outcomes, due to social determinants of these populations, and barriers in access to healthcare services [24].”

Also, this information appears in page 11, lines 225-228: “**** According to official government classification in Colombia, every district is rated from 1 to 6 for its affluence: very low (Status 1), low (Status 2), medium (Status 3), high (Status 4), very high (Status 5), and extremely high (Status 6). Populations living in districts of Status 1 and 2 are considered poor [23].”

**Reviewer #4: The results are clearly presented. the paper has a number of grammatical errors and in two sections the analysis and conclusions do not feel that they are well embedded in the findings. I have made these clear in my accompanying text.**

Authors’ response: Thank you for the suggestions. We have shared the manuscript with an English-native colleague to review grammatical errors. Additionally, we have used an artificial intelligence application to review grammatical errors, avoid overused words, and enhanced the writing. Also, to clarify, we are leaving the quotes as participants stated. We only translated them to English, but leaving the expressions and language style. We have also added some more data in the findings and discussion sections to explain those messages from the conclusion section. Thank you for highlighting it.

**DISCUSION**

**Reviewer #1: (No Response)**

**Reviewer #2:**

**•	You refer the cesarean section as a routine procedure in cases with microcephaly. Can you tell if you have access, the rates of cesarean sections in Colombia?**

Authors’ response: A report published in the Lancet in 2018 by Prof Jane Sandall et al. alerted that Latin America was the region in the world were most C-sections were conducted. In Colombia, C-section rates were reported to be of 45,9%. We have added this publication as a reference in page 25, lines 519-521: “However, caesarean sections should not be routinely performed for the increased risks of maternal morbidity and mortality [48,49]. According to the WHO, these procedures should only represent 10-15% of all deliveries [48,49].”

**•	The syndrome has a significant psychosocial impact on families, especially mothers and consequently health care providers. Can you report similar studies from all the affected areas?**

Authors’ response: Indeed, within our manuscript we cited several studies from other affected areas, conducted with different populations and approaches. Those were conducted by Laza-Vásquez et al. and Romero-Acosta et. al in Colombia; by Kuper et al., Simas et al., Wenham et al., Santos-Pinos et al., Kotzky et al., and Duttine et al. in Brazil; and by Horan et al. in Puerto Rico. Still literature on the psycho-social impacts, and barriers, of mothers of ZIKV-associated microcephalic children is scarce.

**Reviewer #2: CONCLUSIONS**

**•	At this point it is important to emphasize the need to raise awareness of relevant stakeholders, not only locally but also globally. This article highlights not only a significant public health problem and the inequality in access to health services, it presents significant gender inequality issues through presents significant problems of gender inequality through the violation of women's rights and their self-disposition.**

Authors’ response: Thank you for acknowledging it.

**Reviewer #4: There are two conclusions where I feel the authors have drawn conclusions that are not supported by the data. The final sentence of the abstract is also vague and could be far more informative given the richness of the findings.**

Authors’ response: Thank you for acknowledging the richness of the findings. We have re-worded the conclusions to be more informative.

Changes in page 28 lines 575 to 585: “This study contributes to the understanding of the health inequities that ZIKV infection posed on women living in ZIKV affected areas, particularly among those living in areas of low socio-economic statuses, with structural inequalities, such as increased exposure to arboviruses, and lesser access to the healthcare system.”

Changes in page 28 lines 596-598: “It is of high importance to raise awareness of relevant stakeholders locally and globally to respond to all the public health problems including inequalities in access to the healthcare system, gender disparities, and violation of women’s sexual and reproductive rights.”

**GENERAL COMMENTS**

**Reviewer #4: The weakness in the paper include some improvements in grammatical errors made**

Authors’ response: Thank you for this comment. We have improved the writing checking all grammatical errors.

**Reviewer #4: The abstract needs strengthening**

Authors’ response: Thank you for highlighting it. We are following the journal format for displaying the abstract. However, we thoughtfully reviewed the abstract to include more relevant statements and conclusions.

The conclusions of the abstract have been reworded (pages 2 and 3, lines 63-65):

“This study contributed to identifying the social, medical, psychological, and economic needs of families with children affected by the ZIKV epidemics. Commitment and action by local and national governments, and international bodies, is required to ensure sustained and quality health services by affected children and their families.”

**Reviewer #4: The interpretation of the action of health care professionals in relation to abortion, and the comments about the high c section rate stood out as observations that were not substantiated by the data and rather reflected the positionality of the authors.**

Authors’ response:

A report published in the Lancet in 2018 by Prof Jane Sandall et al. alerted that Latin America was the region in the world were most C-sections were conducted. In Colombia, C-section rates were reported to be of 45,9%. We have added this publication as a reference in page 25, lines 519-521: “However, caesarean sections should not be routinely performed for the increased risks of maternal morbidity and mortality [48,49]. According to the WHO, these procedures should only represent 10-15% of all deliveries [48,49].”

Thought the study has a small sample size to draw conclusions on quantitative data, authors found that all interviewed women (N=11) were performed a C-section. According to WHO data, we would expect 1-2 or maybe 3 women to went through a C-section. This finding is not a conclusion based on an epidemiological study, but we understand that it is also a relevant result based on the sample of women interviewed. We have included a sentence in the Results section to highlight this finding in page 9, lines 218-219: “All women gave birth by a C-section”. However, we have reduced the paragraph where it was addressed in the Discussion section (page 25, lines 515-531) to reduce its focus.

Regarding the potential termination of pregnancy, this topic was raised with the participants. There is a full section of results about a “Discussions about a possible abortion” in pages 13-15, lines 299-334. Authors are not showing their positionality about this sensitive topic. We discussed in the manuscript that women would have appreciated having more information about the course of their pregnancy to take their decision based on their sexual and reproductive rights. We also highlight some factors that led women to decide not to terminate their pregnancy, when offered. Those factors include their determination, their families desire, or religious beliefs, among others. Stigma was present in the narratives of those women who decided to continue their pregnancy, but also in those who thought about abortion as a possibility.

**Reviewer #4: These are major revisions that need to be addressed before this important paper can be published.**

Authors’ response: Thank you for this comment. We have worked on improving the quality of the manuscript incorporating all the reviewers’ suggestions.

We incorporated all reviewers’ comments in the manuscript, and have highlighted them in this letter. We hope that this new version meets reviewers’ expectations.

Thank you for your time and constructive feedback.

Authors.
