# Supplementary material for: “One feels anger to know there is no one to help us!”. Perceptions of mothers of children with Zika virus-associated microcephaly in Caribbean Colombia: A qualitative study
Source: PLoS Negl Trop Dis. 2022 Apr 18;16(4):e0010328. doi: 10.1371/journal.pntd.0010328 (PMC9015150; doi:10.1371/journal.pntd.0010328)
Supplement: Attachment — Submitted filename: Reviewer Comments.docx [file pntd.0010328.s006.docx]

Comments

Abstract

Clearly written. I wonder if your concluding statement could be a little more nuanced. ‘Gaps’ and ‘high quality’ don’t tell me anything new. The study design should yield a more meaningful and actionable conclusion.

Be consistent throughout in the style used for direct quotes.

Line 88-89 is confusing. ZIKV infection leads to severe outcomes in children because of its affects to the fetus in utero.

Line 93-94 – is this the case in all settings – that mothers are abandoned - or in Columbia?

Line 97-99 needs to be redrafted.

Line 177 – grammar incorrect – it should be ‘children were’

Tbl 1 – foetus and fetus are not spelled consistently

Line 229 – grammar incorrect

Line 244 – needs rephrasing

Line 266 – missing ‘her’ before mother in law

Line 361 needs rephrasing

Line 410-413 – this needs some rephrasing – do you mean ‘having a child’ not ‘bringing a child’.

Lines 446-448 also needs rephrasing

Line 451-452 – if the women had religious convictions regarding abortion is it not possible that the healthcare workers did too? A description of HCP ‘imposing their social conservative moral values’ feels that it reveals less about the data and more about the interpretation of the authors.

Lines 471-477 reads as a good summary

Lines 497-505 this does not seem to be a sound interpretation of the findings. A small qualitative study is insufficiently powered to say that it reinforces this type of practice.

Line 523 the referencing of Duttine needs to be corrected to follow referencing style

Line 537 replace ‘may not be big’ with ‘was limited’

Researcher positionality
